# Clinical associations of complement-activating collectins, collectin-10, collectin-11 and mannose-binding lectin in preterm neonates

**DOI:** 10.3389/fimmu.2024.1463651

**Published:** 2024-10-11

**Authors:** Gabriela Gajek, Soren W. K. Hansen, Dariusz Jarych, Maja Kufelnicka-Babout, Anna S. Świerzko, Paulina Kobiela, Agnieszka Szala-Poździej, Karolina Chojnacka, Katarzyna Sobczuk, Iwona Domżalska-Popadiuk, Jan Mazela, Jarosław Kalinka, Steffen Thiel, Maciej Cedzyński

**Affiliations:** ^1^ Laboratory of Immunobiology of Infections, Institute of Medical Biology, Polish Academy of Sciences, Łódź, Poland; ^2^ Department of Cancer and Inflammation Research, Institute of Molecular Medicine, University of Southern Denmark, Odense, Denmark; ^3^ Department of Perinatology, First Chair of Gynecology and Obstetrics, Medical University of Łódź, Łódź, Poland; ^4^ Department of Neonatology, Medical University of Gdańsk, Gdańsk, Poland; ^5^ II Department of Neonatology, Poznań University of Medical Sciences, Poznań, Poland; ^6^ Department of Neonatology, Poznań University of Medical Sciences, Poznań, Poland; ^7^ Department of Biomedicine, Aarhus University, Aarhus, Denmark

**Keywords:** CL-10, CL-11, collectin, complement, mannose-binding lectin (MBL), neonate, prematurity, respiratory distress syndrome (RDS)

## Abstract

**Introduction:**

Premature and low-birthweight infants are at especially high risk of perinatal complications, including impaired thermoregulation, infections and respiratory distress. Such adverse effects and the need for invasive procedures are associated with high mortality among preterms. This study focused on the influence of the innate immune system and tested the levels of collectins, collectin-10 (CL-10), collectin-11 (CL-11) and mannose-binding lectin (MBL) in preterm neonates.

**Methods:**

Cord blood was collected from 535 preterms (born at gestational age ≤37 weeks). *COLEC10* and *COLEC11* polymorphisms were analyzed by real-time PCR and those of *MBL2* by PCR/PCR-RFLP. The concentrations of collectins in sera from cord blood were determined with ELISA.

**Findings:**

Low concentrations of CL-10 in cord sera (<462 ng/ml corresponding to the 10^th^ percentile) were significantly associated with births at GA ≤32 weeks. Median levels of both CL-10 and CL-11 were significantly lower in preterms with very low birthweight (<1500 g), low Apgar 1’ score and those who needed prolonged hospitalisation. Lower median CL-10 was also observed in fetal growth restriction cases. An important finding was the decreased concentrations of CL-10, CL-11 and MBL in respiratory distress syndrome (RDS). For CL-10 and CL-11, that relationship was confined to infants born at GA ≥33 weeks and/or with body mass at birth ≥1500 g. Only CL-10 was found to influence susceptibility to early-onset infections. *COLEC11* heterozygosity for the activity-decreasing polymorphism (rs7567833, +39618 A>G, His219Arg) was more common in preterm premature rupture of membranes (pPROM) cases, compared with corresponding reference groups. Furthermore, C/T or T/T genotypes at *COLEC11* at rs3820897 (-9570 C>T) as well as MBL deficiency-associated *MBL2* gene variants were more common in preterms diagnosed with RDS than among unaffected newborns.

**Conclusion:**

The complement-activating collectins investigated here could be important for maintaining homeostasis in preterm neonates. Despite similar structure and specificity, MBL, CL-10 and CL-11 manifest a different spectrum of clinical associations.

## 1 Introduction

Approximately 11% of babies globally are born preterm (before 37 weeks) (1). In 2022, 7.2% of live births in Poland were premature. Among 305,132 newborns, 2,453 (0.8%) had body mass <1500 g at birth (2). Premature and low-birthweight babies are at especially high risk of perinatal complications, including impaired thermoregulation, infections and respiratory distress. Those complications and the need for invasive procedures are associated with relatively high mortality among preterms: 65.1% of infants who died aged ≤1 year (764/1171) were born prematurely. Furthermore, as many as 40.2% (321/798) of neonatal deaths were associated with birthweight (BW) <1500 g (2). That reflected rather underdevelopment of the respiratory system and other adverse effects of prematurity than low body mass itself. Additionally, perinatal complications may affect the quality of life during childhood and even adulthood (1, 3, 4).

The neonatal immune system is immature, especially in preterm newborns. Furthermore, premature delivery abrogates the transfer of maternal immunoglobulins through the placenta and thus does not allow them to reach a sufficient concentration in the infant’s blood. The lack of “immune experience” (exposure to “non-self” antigens) is reflected by a lack of specific T and B lymphocytes and, therefore, impaired cellular and humoral response. Low level or lack of antibodies recognising polysaccharide or glycoconjugate antigens may, at least partially, be compensated by soluble defence lectins, including collectins (5, 6).

Collectins are oligomeric C-type defence lectins. As pattern-recognising molecules (PRMs), they contribute to maintaining homeostasis by elimination of pathogens and altered self-cells by opsonisation or agglutination. Three of these PRMs, mannose-binding lectin (or mannan-binding lectin, MBL), collectin-10 (CL-10; or collectin liver-1, CL-L1) and collectin-11 (CL-11; collectin kidney-1, CL-K1) form complexes with MASPs (MBL-associated serine proteases), enabling activation of complement *via* the lectin pathway (LP). Those lectins recognise a variety of carbohydrate residues, including D-mannose, N-acetyl-D-glucosamine and L-fucose, that commonly decorate microbial surface structures [reviewed in (5, 7, 8)]. The ligand specificity of CL-10 and CL-11 may be extended by forming their heterocomplexes called CL-LK (9). Moreover, the interaction of CL-11 with C1q/TNF-related protein 6 (CTRP-6) enables its specific recruitment to molecular conserved motifs, common for a variety of microbes (pathogen-associated molecular patterns, PAMP) or endogenous signals released following host’s cell damage or death (danger-associated molecular patterns, DAMP), followed by complement activation (10). As a part of the innate immune system, the mentioned collectins are important players in neonatal response to the extrauterine conditions. It should, however, be stressed that they may not only protect from infection but also participate in pathophysiological processes, including SIRS/sepsis (5).

Certain known, rare mutations of corresponding *COLEC10* and *COLEC11* genes were found to cause Malpuech facial clefting syndrome (3MC syndrome), with craniofacial, renal or genital abnormalities, growth and intellectual disability [reviewed in (11)]. In contrast, MBL deficiency is considered the most frequent human immunodeficiency. It is associated with several single nucleotide polymorphisms of the *MBL2* gene, localised to the promoter region and/or exon 1: -550 G>C (rs11003125, commonly termed H/L), -221 G>C (rs76206, Y/X), C4T (rs7095891, P/Q), C223T (R52C, rs5030737, A/D), G230A (G54D, rs1800450, A/B) and G239A (G57E, rs1800451, A/C). Variant alleles D, B, and C of exon 1 coding region are collectively called O. The MBL inborn deficiency is generally associated with O/O and LXPA/O (or LXA/O, XA/O) genotypes [reviewed in (5)].

The ficolin family is another group of PRMs and is structurally related to collectins by possessing a collagen-like region, but in this case, with fibrinogen-like domain as recognition module. Recently, we reported associations of the *FCN2* gene polymorphisms regions and concentrations of its product, ficolin-2, in cord sera with clinical features of Polish preterm neonates (12–14). Here we extend those data with results concerning relationships between CL-10, CL-11, MBL, and selected clinical parameters as well as complications commonly associated with prematurity. Although numerous reports concerning the role of MBL in neonatal health and disease have been published, data are often inconsistent while the literature concerning CL-10 and CL-11 is still rather scarce and focused mainly on their crucial role in fetal development (5). Therefore, the aim of our study was to determine relationships between the mentioned complement-activating collectins and perinatal, prematurity-associated complications, including early-onset infections, respiratory distress, fetal growth restriction, preterm premature rupture of membranes and need for intensive care. To achieve those goals, concentrations of CL-10, CL-11 and MBL in cord sera as well as selected polymorphisms of corresponding genes were investigated in a relatively large cohort of preterm neonates. Additionally, as no data concerning associations of CL-10 or CL-11 with gestational age have been previously published, we tested a series of cord serum samples from term deliveries and determined expression of the *COLEC10, COLEC11* and *MBL2* in preterm and term placentas.

## 2 Materials and methods

### 2.1 Subjects

Cord blood samples from 535 preterm newborns (born between 24^th^ and 37^th^ weeks of pregnancy), including 112 born at gestational age (GA) <33 weeks, were obtained from the Department of Newborns’ Infectious Diseases (Poznań University of Medical Sciences, Poland) (n=272), Department of Neonatology (Medical University of Gdańsk, Poland) (n=207) and Department of Perinatology (Medical University of Łódź, Poland) (n=56). This cohort included 504 babies previously investigated by us (12–14). Three hundred and forty two individuals came from singleton pregnancies, 187 from 106 twin pregnancies and 6 from 2 triple pregnancies. From 25 twin pairs, material from only one sibling was collected. Furthermore, cord blood samples from randomly selected 17 term neonates (GA 38-41 weeks, no symptoms of infection within 72 h after birth) and sections of placenta from randomly selected 35 live births (GA 30-41 weeks) were collected (Medical University of Łódź, Poland). The exclusion criteria included maternal COVID-19, HIV infection and viral hepatitis. The study was approved by the corresponding local ethics committees (Bioethics Committee of Poznań University of Medical Sciences, Independent Bioethics Committee for Scientific Research at Medical University of Gdańsk, Bioethics Committee of The Medical University of Łódź). Written informed parental consent was obtained. This work conforms to the provisions of the Declaration of Helsinki.

### 2.2 DNA, serum and mRNA samples

Cord blood samples for DNA extraction were taken into S-Monovette citrated tubes (Sarstedt, Germany) and stored at -20 °C. DNA was extracted with the use of GeneMATRIX Quick Blood DNA Purification Kit (EURx Ltd, Poland), according to the manufacturer’s protocol. Total RNA was isolated from placenta sections, using the GeneMATRIX Universal RNA purification kit (EURx Ltd) according to the manufacturer’s protocol. DNA and RNA concentrations were measured using a NanoDrop™ 2000 spectrophotometer (Thermo Fisher Scientific, USA). The absorbance 260/280 nm and 260/230 nm ratios were evaluated to control residual contamination from the nucleic acids extraction procedure. Samples for serum isolation were taken into S-Monovette tubes with Z-Gel clot activator (Sarstedt). Sera were stored at -80°C until tested.

### 2.3 Genotyping

Single nucleotide polymorphisms (SNP) of the *MBL2* gene, localised to promoter (H/L, at position -550, rs11003125 and Y/X, at position -221, rs7096206) were analysed using allele-specific PCR, previously described (n=535) (14). Exon 1 (A/D, codon 52, rs5030737; A/B, codon 54, rs1800450 and A/C, codon 57, rs1800451) polymorphisms were investigated with the use of PCR-RFLP procedures, employing MluI, BshNI and MboII (all purchased from Thermo Fisher Scientific) enzymes, respectively (15). As mentioned above, those polymorphisms affect MBL expression and/or activity and LXA/O or O/O genotypes are associated with its primary deficiency.

*COLEC10* and *COLEC11* polymorphisms were analyzed using TaqMan^®^ SNP Genotyping Assays (Thermo Fisher Scientific). For *COLEC10*, rs149331285 (Assay ID: C_174416274_10) and rs148350292 (custom TaqMan probe), were genotyped. For *COLEC11*, rs3820897 (Assay ID: C:_2040704_10) and rs7567833 (Assay ID: C:25989885_10) were investigated (n=420). The reactions were conducted in a final volume of 20 µl on QuantStudio™ 5 Real-Time PCR System (Applied Biosystems by Thermo Fisher Scientific), using a 2x TaqMan™ Genotyping Master Mix. The reaction conditions were as follows: 95°C for 10 minutes, followed by 40 cycles of 95°C for 15 seconds and 60°C for 1 minute. The results were analyzed using the QuantStudio™ Design & Analysis Software v1.5.1” (Thermo Fisher Scientific). *COLEC10* exon 5 rs149331285 (+36545 T>C, Arg125Trp) was previously found to affect concentration of CL-10 in serum whilst promoter five-nucleotide deletion rs148350292 (−161_−157 delAAAAT) overlaps with transcription factors binding sites, affecting developmental processes (including liver differentiation) (16). *COLEC11* exon rs7567833 (+39618 A>G, His219Arg) was previously found not to influence CL-11 level but it was chosen as a non-synonymous polymorphism, affecting protein structure. Carrying of the promoter rs3820897 (-9570 C>T) variant allele was, in turn, demonstrated to be associated with a lower concentration of this lectin in serum (16).

### 2.4 Determination of concentrations of collectins in cord serum samples

CL-10 and -11 concentrations (in cord sera from 420 preterm and 17 term neonates) were determined in ELISA, as described by Axelgaard et al. (17) and Selman et al. (18), respectively. Briefly, MaxiSorp F96 immunoplates (NUNC, Denmark) were coated with murine anti-human CL-10 or CL-11 antibodies (clones 16-1 and 11-10, respectively, 5 µg/ml), in coating buffer (15 mM Na_2_CO_3_, 35 mM NaHCO_3_, pH 9,6). Plates were incubated overnight at 4°C. After blocking with TBS (10 mM Tris, 145 mM NaCl, pH 7.4) supplemented with 5 mM EDTA and 0.05% Tween (pH 7.4) and incubation with tested sera (prediluted 1:50 in sample buffer: TBS, supplemented with 5 mM EDTA, 0.05% Tween, 0.1% bovine serum and 50 µg/ml heat-aggregated human IgG), the bound proteins were detected with the help of biotinylated anti-CL-10 (clone 16-13) or anti-CL-11 (clone 16-25) (0.5 µg/ml) antibodies (diluted in TBS supplemented in 5 mM EDTA, 0,05% Tween and 1 mg/ml BSA) and HRP-labelled streptavidin (0.1 µg/ml in TBS supplemented with 5 mM EDTA, 0.05% Tween and 1 mg/ml BSA). Next, TMB One was added to each well, and the plate was incubated for 15 min in a dark chamber. A standard curve was constructed from serially diluted standard serum. For negative control, the addition of serum was omitted. CL-10 concentrations <462 ng/ml and CL-11 levels <223 ng (<10^th^ percentile determined for preterms) were considered low.

The concentration of MBL in cord serum samples (n=448) was determined by ELISA based on binding to solid-phase yeast mannan (Sigma-Aldrich) and detection with anti-human MBL mAb (HYB131-1, Statens Serum Institut, Denmark) as described by Cedzynski et al. (19). Concentrations <150 ng/ml were considered low.

### 2.5 Expression of genes in placenta

The cDNA was obtained using High-Capacity cDNA Reverse Transcription Kit (Applied Biosystems, Thermo Fisher Scientific). The expression of mRNA from *COLEC10*, *COLEC11*, as well as two housekeeping genes *YWHAZ* and *SDHA* (20), were analyzed using the primers listed in **Table 1**. Expression of mRNA from the *MBL2* gene was analysed with the use of commercial primers provided by Qiagen (PPH07136E).

**Table 1:** Sequences of primers used for investigation of *COLEC10, COLEC11* and housekeeping *YWHAZ* and *SDHA* genes.

Gene	Primer sequence:	
***COLEC11***	Forward	5’-CTAGCGCGTGCTCAGGAGTT-3’
	Reverse	5’-CGGCGACAGCATTCTTGATG-3’
***COLEC10***	Forward	5’-AGCCGTCCTACCGCTGAAGT-3’
	Reverse	5’-GCGTCCCACTTTGCCATGCT-3’
***YWHAZ***	Forward	5’-ACTTTTGGTACATTGTGGCTTCA-3’
	Reverse	5’-CCGCCAGGACAAACCAGTAT-3’
***SDHA***	Forward	5’-TGGGAACAAGAGGGCATCTG-3’
	Reverse	5’-CCACCACTGCATCAAATTCATG-3’

The PCR mixture consisted of 10 µL of 2x miRCURY LNA SYBR Green PCR Kit (Qiagen), 1 µl of appropriate primers and 1 µL of cDNA template. The analyses were carried out using the QuantStudio™7 (Applied Biosystems, Thermo Fisher Scientific). All reactions were performed under the following conditions: an initial denaturation step at 95°C for 10 min, followed by 40 amplification cycles of denaturation (95°C, 15 s), a single annealing and extension step (60°C for 1 min), in duplicates.

### 2.6 Statistical analysis

The frequencies of genotypes were compared by Fischer’s exact test (two-tailed) or χ^2^ test when appropriate. The Shapiro-Wilk test was used for the determination of normality of distributions of gestational age, birthweight as well as CL-10, CL-11 and MBL concentrations. The median levels of collectins were compared with Mann-Whitney *U* test with Bonferroni correction or Kruskal-Wallis test with *post-hoc* Dunn’s test when appropriate. For comparisons between twins, Wilcoxon matched-pairs test was employed. For correlations, Spearman’s coefficients were calculated. For multiple logistic regression analysis, each of the following parameters or clinical conditions: gestational age <33 weeks, birthweight <1500 g, 1’ Apgar score <7, hospitalization >14 days, NICU stay >4 days, need for respiratory support, respiratory distress syndrome (RDS), fetal growth restriction (FGR), preterm premature rupture of membranes (pPROM), early-onset infection (EOI), pneumonia was analyzed as dependent variable with the use of the following set of independent variables: low (<462 ng/ml) CL-10, low (<223 ng/ml) CL-11, low (<150 ng/ml) MBL concentrations, presence of variant alleles for *COLEC11* rs3820897 and rs7567833, MBL deficiency-associated genotype. The Statistica (version 13.3, TIBCO Software) and SigmaPlot (version 12, Systat Software) software packages were used for data management and statistical calculations. Odds ratio was calculated using online MedCalc software (https://www.medcalc.org). P values <0.05 were considered statistically significant.

## 3 Results

### 3.1 Basic data

Both gestational age (GA) and birthweight (BW) were not normally distributed (medians: 35 weeks and 2320 g, respectively). As expected, the GA and BW were highly correlated (r=0.687, p<0.000001) (**Supplementary Figure 1**).

Polymorphisms of the *COLEC10* and *COLEC11* genes as well as serum concentrations of CL-10 and CL-11 were investigated in 420 preterm infants. As genotyping failed in 6 cases for *COLEC10* and in 5 instances for *COLEC11*, therefore data from 414 and 415 individuals were available for analyses of corresponding polymorphisms, respectively. For *COLEC10*, all babies were major (C) allele homozygotes at rs149331285 and only 2 heterozygotes were found at rs148350292. These polymorphisms were, therefore, not submitted for further statistical analysis. For *COLEC11* rs3820897, 287 (69.2%) neonates had C/C, 107 (25.8%) had C/T and 21 (5.1%) had T/T genotypes, respectively. Analysis of rs7567833, revealed 382 (91.8%) A/A homozygotes and 34 (8.2%) A/G heterozygotes. No variant homozygote was found.

The median serum CL-10 level equalled 667 ng/ml, while that of CL-11 was 321 ng/ml. CL-10 concentrations in cord sera ranged from 172 ng/ml to 2273 ng/ml (IQR: 559-888 ng/ml). Corresponding values for CL-11 were found to be 78-1306 ng/ml and 270-402 ng/ml, respectively. Levels <10^th^ percentile (462 ng/ml and 223 ng/ml for CL-10 and CL-11, respectively) were considered low.

No significant differences in CL-11 concentrations were found among carriers of different *COLEC11* genotypes at rs7567833 (+39618 A>G, His219Arg) or rs3820897 (-9570 C>T) (**Supplementary Figures 2A, B**, respectively). The concentrations of CL-10 and CL-11 were highly correlated (r=0.912, p<0.000001) (**Supplementary Figure 3A**). Furthermore, the levels of both collectins correlated weakly with ficolin-2 [data recently reported by Gajek et al. (14)]: r=0.129, p=0.0098 (**Supplementary Figure 3D**) and r=0.123, p=0.014 (**Supplementary Figure 3E**), respectively.

Among 535 premature newborns tested for *MBL2* gene polymorphisms, 352 (65.8%) had A/A genotypes, 162 (30.3%) were A/O heterozygotes, while 21 (3.9%) were O/O homo- or heterozygotes. In 40 A/O heterozygotes, the A allele was combined with the LX promoter haplotype; therefore, 61 (11.4%) altogether were considered MBL-deficient (LXA/O or O/O genotype).

Mannose-binding lectin concentrations were determined in cord serum samples from 448 babies. The median MBL concentration was 642 ng/ml (range: 0-3955 ng/ml, interquartile range (IQR): 183-1153 ng/ml). Ninety-four (21%) individuals had low (<150 ng/ml) concentrations. Typically, the highest median corresponded to A/A (903 ng/ml), while the lowest corresponded to XA/O and O/O genotypes (39 ng/ml), respectively (p<0.000001). No significant correlations with CL-10, (**Supplementary Figure 3B**), or CL-11 (**Supplementary Figure 3C**) or ficolin-2 (**Supplementary Figure 3F**) levels were observed (r<0.1).

As the concentrations of none of those serum collectins were normally distributed (not shown), non-parametric tests were used for statistical calculations.

### 3.2 Associations of collectins with gestational age and birthweight

The concentrations of CL-10, CL-11 and MBL correlated weakly but significantly with gestational age [r=0.175, p=0.00035 (**Supplementary Figure 4A**); r=0.13, p=0.0081 (**Supplementary Figure 4B**); r=0.114, p=0.015 (**Supplementary Figure 4C**), respectively]. Since, to our knowledge, no reports concerning associations of CL-10 or CL-11 with GA have been published, we measured their concentrations (as well as MBL levels) in cord serum samples from 17 term babies (GA 38-41 weeks). As expected, those relationships were confirmed (r=0.164, p=0.00064; r=0.145, p=0.0026; r=0.13, p=0.005 for CL-10, CL-11 and MBL, respectively).

**Figure 1:**
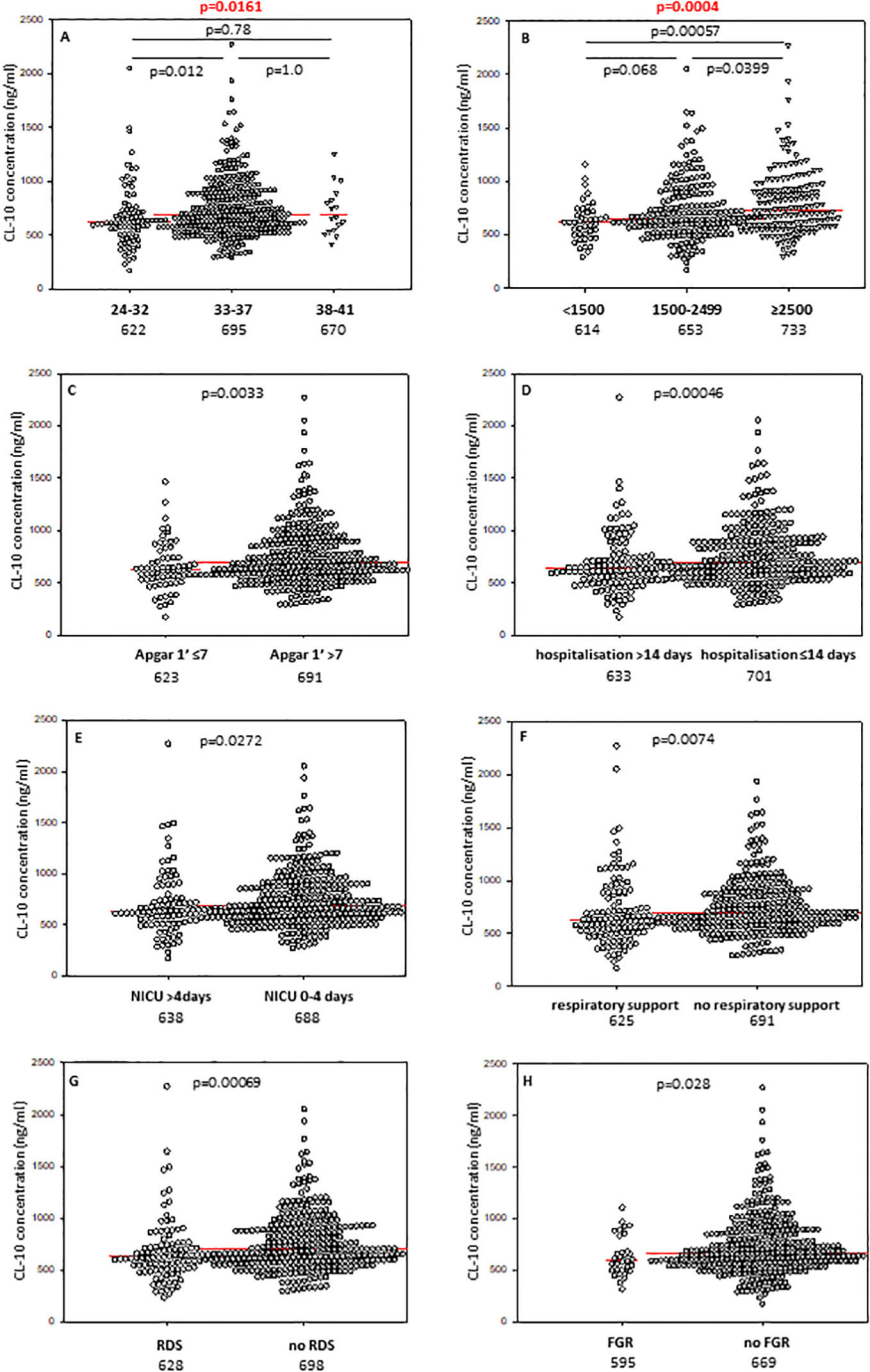
Individual concentrations of collectin-10, depending on gestational age **(A)**, birthweight **(B)**, 1’ Apgar score **(C)**, length of hospitalisation **(D)**, length of intensive care **(E)**, need for respiratory support **(F)**, incidence of respiratory distress syndrome **(G)**, and fetal growth restriction **(H)**. Red bars represent median values (demonstrated as numbers below the graphs). Statistical significance values show comparisons with the use of Kruskal-Wallis test with *post hoc* Dunn test **(A, B)** or Mann-Whitney *U* test **(C–H)**.

Babies born at gestational age (GA) ≤32 weeks had lower CL-10 concentrations compared with those born at GA 33-37 weeks but not from term newborns, probably due to the low number of subjects in the last mentioned group (**Figure 1A**). However, Kruskal-Wallis test demonstrated a significant association between gestational age and CL-10 concentration (**Figure 1A**). The frequency of low values differed significantly between babies born at GA ≤32 and 33-37 weeks: 17/88 (19.3%) *vs*. 25/325 (7.7%). The statistical analysis is summarised in **Table 2**.

**Table 2:** Univariate analysis of associations of low concentrations of CL-10, CL-11 and MBL and selected polymorphisms of *COLEC11* and *MBL2* genes with clinical parameters and perinatal complications in preterm neonates (the significant associations are marked in red).

Clinical parameter/complication	Genotype/low collectin concentration in cord serum					
	Low CL-10 (<462 ng/ml *vs.* ≥462 ng/ml)	Low CL-11 (<223 ng/ml *vs.* ≥223 *n*g/ml)	*COLEC11* rs3820897 (C/T or T/T *vs.* C/C)	*COLEC11* rs7567833 A/G *vs.* A/A	Low MBL (<150 ng/ml *vs.* ≥150 ng/ml)	MBL primary deficiency (*MBL2* (XA/O or O/O *vs.* A/A or YA/O)
**GA <33 weeks** ***vs.* ≥33 weeks**	p=0.026 OR=2.87 (1.47-5.61)	p=0.0041 OR=2.8 (1.41-5.54)	p=0.79 OR=1.08 (0.65-1.8)	p=1.0 OR=0.98 (0.41-2.33)	p=0.57 OR=1.2 (0.7-2.06)	p=0.55 OR=0.81 (0.41-1.62)
**BW <1500 g** ***vs*. ≥2500 g**	p=0.0091 OR=4.05 (1.46-11.26)	p=0.0049 OR=4.23 (1.59-11.22)	p=0.26 OR=0.93 (0.45-1.93)	p=1.0 OR=1.11 (0.29-4.16)	p=0.22 OR=1.68 (0.8-3.55)	p=0.64 OR=0.73 (0.26-2.01)
**1’ Apgar** **score <7** ***vs*. ≥7**	p=0.27 OR=1.56 (0.7-3.45)	p=0.25 OR=1.62 (0.73-3.6)	p=0.1 OR=1.52 (0.95-2.45)	p=0.68 OR=1.2 (0.55-2.61)	p=1.0 OR=1.0 (0.52-1.9)	p=1.0 OR=1.01 (0.45-2.24)
**Hospitalisation** **>14 days** ***vs.* 0-14 days**	p=0.045 OR=2.02 (1.05-3.9)	p=0.011 OR=2.4 (1.23-4.66)	p=0.25 OR=1.32 (0.85-2.06)	p=0.85 OR=1.1 (0.52-2.29)	p=1.0 OR=0.99 (0.61-1.61)	p=0.77 OR=1.09 (0.61-1.94)
**NICU stay** **>4 days** ***vs.* 0-4 days**	p=0.048 OR=2.09 (1.05-4.15)	p=0.16 OR=1.7 (0.84-3.46)	p=0.37 OR=1.28 (0.79-2.09)	p=0.67 OR=1.21 (0.55-2.69)	p=0.5 OR=1.2 (0.71-2.02)	p=0.23 OR=1.44 (0.8-2.62)
**Respiratory support** ***vs.* no support**	p=0.0019 OR=2.85 (1.48-5.48)	p=0.01 OR=2.41 (1.24-4.66)	p=0.046 OR=1.59 (1.01-2.49)	p=0.84 OR=1.04 (0.48-2.25)	p=0.44 OR=1.21 (0.74-1.99)	p=0.79 OR=0.92 (0.51-1.61)
**RDS** ***vs.* no RDS**	p=0.01 OR=2.46 (1.26-4.82)	p=0.051 OR=2.04 (1.02-4.05)	p=0.019 OR=1.76 (1.1-2.81)	p=0.68 OR=0.78 (0.33-1.86)	p=0.077 OR=1.59 (0.96-2.64)	p=0.059 OR=1.72 (0.97-3.05)
**FGR** ***vs.* no FGR**	p=0.35 OR=1.71 (0.62-4.72)	p=0.54 OR=1.37 (0.45-4.12)	p=0.24 OR=1.56 (0.74-3.27)	p=0.73 OR=1.25 (0.36-4.34)	p=1.0 OR=1.0 (0.48-2.09)	p=0.39 OR=1.4 (0.65-3.0)
**pPROM** ***vs.* no pPROM**	p=0.67 OR=1.23 (0.56-2.7)	p=0.83 OR=1.1 (0.49-2.49)	p=0.59 OR=1.67 (0.99-2.81)	p=0.039 OR=2.28 (1.06-4.89)	p=0.89 OR=1.04 (0.59-1.83)	p=0.88 OR=1.05 (0.54-2.06)
**EOI** ***vs.* no EOI**	p=0.17 OR=1.84 (0.86-3.97)	p=1.0 OR=0.94 (0.18-2.33)	p=0.77 OR=1.09 (0.62-1.91)	p=0.46 OR=1.43 (0.6-3.45)	p=0.29 OR=1.25 (0.67-2.32)	p=0.43 OR=0.67 (0.27-1.62)
**Pneumonia** ***vs.*** **no pneumonia**	p=0.43 OR=1.45 (0.53-3.94)	p=0.77 OR=1.13 (0.38-3.36)	p=0.58 OR=1.7 (0.84-3.45)	p=0.76 OR=0.62 (0.14-2.68)	p=0.41 OR=1.38 (0.64-2.95)	p=0.097 OR=0.32 (0.07-1.33)

**Figure 2:**
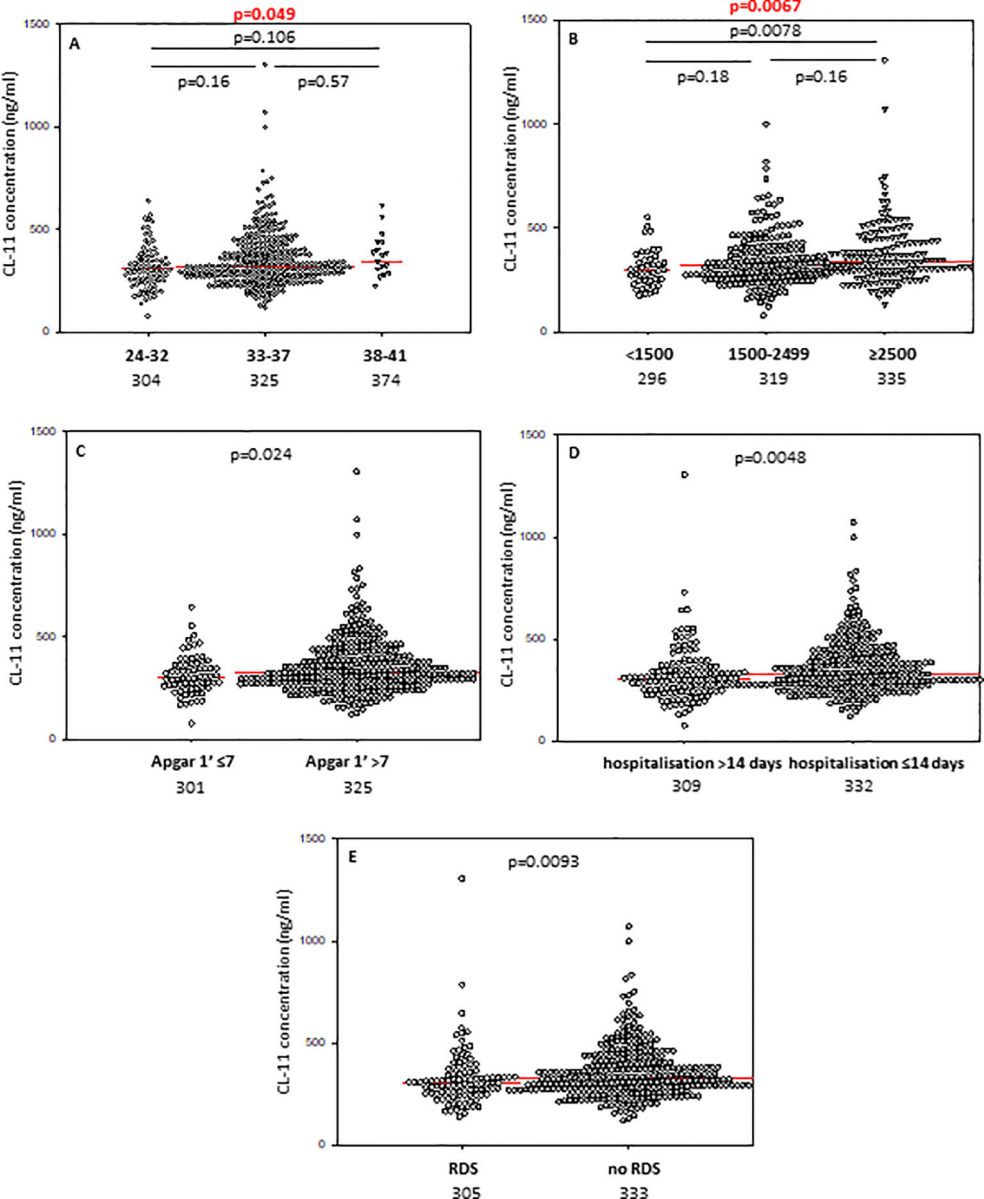
Individual concentrations of collectin-11, depending on gestational age **(A)**, birthweight **(B)**, 1’ Apgar score **(C)**, length of hospitalisation **(D)**, and incidence of respiratory distress syndrome **(E)**. Red bars represent median values (demonstrated as numbers below the graphs). Statistical significance values show comparisons with the use of Kruskal-Wallis test with *post hoc* Dunn test **(A, B)** or Mann-Whitney *U* test **(C–E)**.

In contrast to CL-10, the median CL-11 level determined for preterms with shorter GA was significantly lower compared with term babies but not with preterms born at GA 33-37 weeks (**Figure 2A**). When Kruskal-Wallis test was performed, the median CL-11 concentrations in cord sera were significantly different in regard to GA (**Figure 2A**). Low concentrations were found more frequent among neonates born at ≤32 GA compared with those born at GA 33-37 weeks: 16/88 (18.2%) *vs*. 24/326 (7.4%) (**Table 2**).

**Figure 3:**
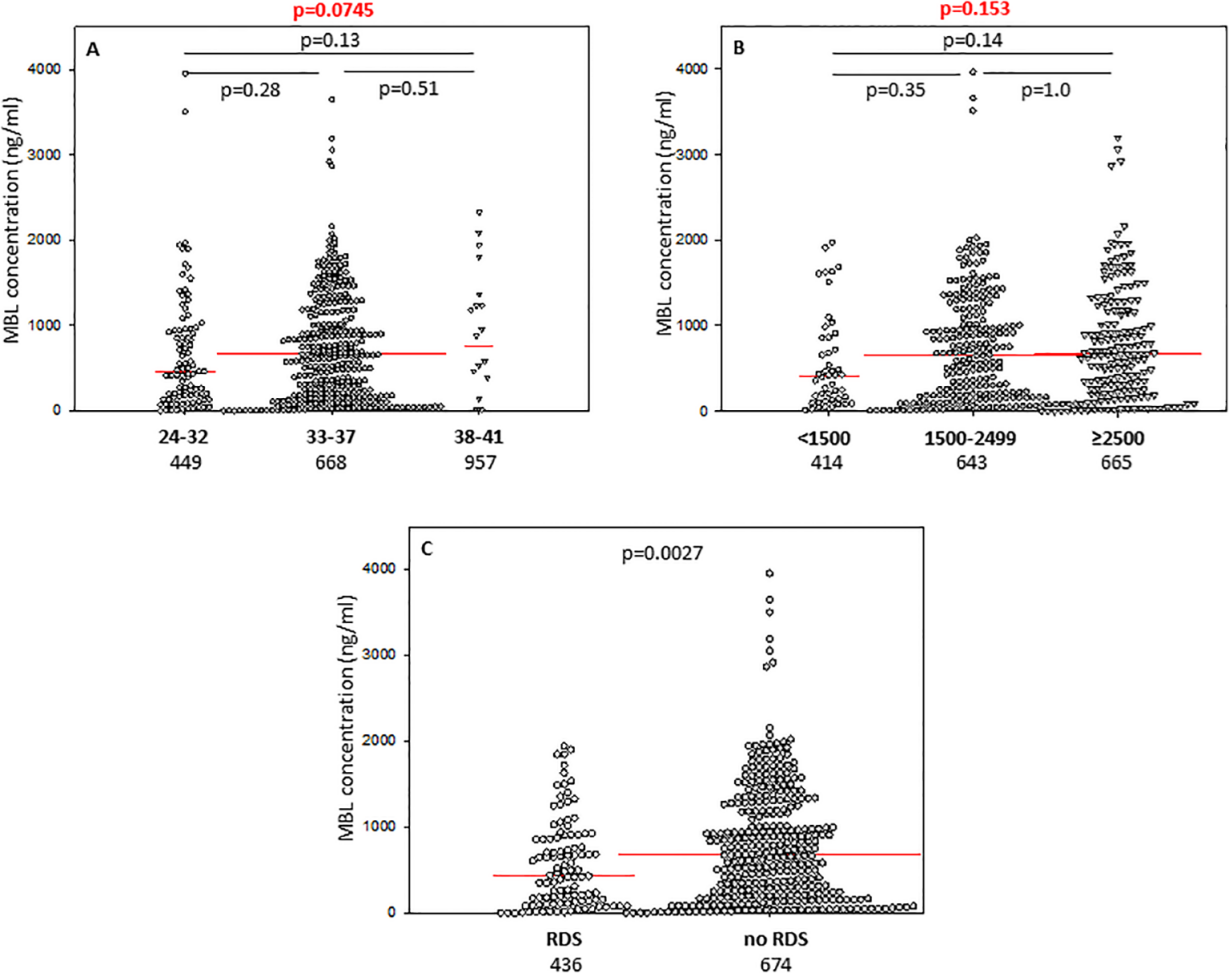
Individual concentrations of mannose-binding lectin, depending on gestational age **(A)**, birthweight **(B)**, and incidence of respiratory distress syndrome **(C)**. Red bars represent median values (demonstrated as numbers below the graphs). Statistical significance values show comparisons with the use of Kruskal-Wallis test with *post hoc* Dunn test **(A, B)** or Mann-Whitney *U* test **(C)**.

The difference between median serum MBL in babies born at GA 24-32 and those born at GA 33-37 did not reach statistical significance (**Figure 3A**), nor was there any significant difference in frequency of low (<150 ng/ml) concentrations between those groups: 22/94 (24.5%) *vs.* 72/354 (20.3%) (**Table 2**).

The frequency of MBL deficiency-associated genotypes (LXA/O plus O/O) was similar in both preterm groups [11/112 (9.8%) *vs.* 50/423 (11.8%), **Table 2**]. Similarly, the numbers of carriers of *COLEC11* C/T or C/C (rs3820897) and A/G (rs7567833) genotypes barely differed between babies born at GA ≤32 and ≥33 weeks [28/87 (32.2%) *vs.* 100/328 (30.5%), and 7/87 (8%) *vs.* 27/329 (8.2%), p=1.0, respectively (**Table 2**).

The concentrations of CL-10 in cord sera correlated significantly with birthweight in premature babies born at GA 24-37 weeks (r=0.218, p=0.000008) (**Supplementary Figure 4D**). Similar associations were found for CL-11 (r=0.169, p=0.00058) (**Supplementary Figure 4E**) but not MBL (r=0.062, p=0.19) (**Supplementary Figure 4F**). Corresponding differences in median levels of CL-10 and CL-11 were apparent when preterm neonates were classified into low (<1500 g), medium (1500g - 2499 g) and high (≥2500 g) BW groups. For MBL, median values from babies born with the lowest BW differed significantly from those of the highest BW. Individual concentrations, of tested collectins, depending on birthweight ranges are presented in **Figure 1B** (CL-10), **Figure  2B** (CL-11) and **Figure 3B** (MBL).

The frequency of low levels of collectin-10 among newborns with body mass at birth <1500 g (8/42, 19%) was significantly higher compared with that among newborns with BW ≥2500 g (9/164, 5.5%) (**Table 2**). CL-10 concentrations <10^th^ percentile were also found in 23/204 (11.3%) of babies born with BW 1500-2499 g (no significant difference in comparison with afore-mentioned groups).

The lowest CL-11 levels in cord sera were observed in 9/42 (21.4%), 19/204 (9.3%) and 10/165 (6.1%) neonates, depending on the BW groups as defined. The differences were statistically significant between the low and high BW groups (**Table 2**). Moreover, low CL-11 was found more frequently among preterms with BW <1500 g than among those with BW 1500-2499 g [p=0.033, OR=2.66, 95% CI (1.11-6.37)]. No significant difference in the frequency of CL-11 concentrations <223 ng/ml was found between the medium and the high BW groups (p=0.33). However, multiple logistic regression analysis demonstrated an association of low CL-11 in cord serum with low body mass at birth (OR>3.5) (**Table 3**).

**Table 3:** Multiple logistic regression analysis of associations of low concentrations of CL-10, CL-11 and MBL and selected polymorphisms of *COLEC11* and *MBL2* genes with clinical parameters and perinatal complications in preterm neonates (the significant associations are marked in red).

Dependent variables	Independent variables					
	Low CL-10 (<462 ng/ml *vs.* ≥462 ng/ml)	Low CL-11 (<223 ng/ml *vs.* ≥223 *n*g/ml)	*COLEC11* rs3820897 (C/T or T/T *vs.* C/C)	*COLEC11* rs7567833 A/G *vs.* A/A	Low MBL (<150 ng/ml *vs.* ≥150 ng/ml)	MBL primary deficiency (*MBL2* (XA/O or O/O *vs.* A/A or YA/O)
**GA <33 weeks**	p=0.37 OR=1.55 (0.6-3.99)	p=0.11 OR=2.2 (0.84-5.78)	p=0.75 OR=1.09 (0.63-1.88)	p=0.88 OR=1.07 (0.44-2.62)	p=0.28 OR=1.45 (0.74-2.82)	p=0.51 OR=0.75 (0.32-1.77)
**BW <1500 g**	p=0.91 OR=0.93 (0.26-3.33)	p=0.04 OR=3.55 (1.06-11.92)	p=0.91 OR=0.96 (0.45-2.03)	p=0.93 OR=0.95 (0.27-3.34)	p=0.29 OR=1.6 (0.67-3.84)	p=0.52 OR=0.68 (0.21-2.22)
**1’ Apgar** **score <7**	p=0.95 OR=1.04 (0.33-3.25)	p=0.3 OR=1.82 (0.59-5.63)	p=0.043 OR=1.81 (1.02-3.2)	p=0.42 OR=1.45 (0.59-3.54)	p=0.54 OR=1.27 (0.59-2.7)	p=0.8 OR=0.88 (0.34-2.31)
**Hospitalisation** **>14 days**	p=0.67 OR=1.21 (0.5-2.97)	p=0.057 OR=2.43 (0.97-6.06)	p=0.65 OR=1.12 (0.7-1.78)	p=0.88 OR=0.94 (0.43-2.07)	p=0.97 OR=0.99 (0.54-1.82)	p=0.89 OR=1.05 (0.5-2.21)
**NICU stay** **>4 days**	p=0.028 OR=2.86 (1.12-7.34)	p=0.68 OR=0.8 (0.29-2.25)	p=0.39 OR=1.26 (0.75-2.12)	p=0.48 OR=1.35 (0.59-3.06)	p=0.44 OR=1.29 (0.68-2.48)	p=0.81 OR=1.1 (0.5-2.43)
**Respiratory support**	p=0.005 OR=3.72 (1.5-9.27)	p=0.89 OR=0.93 (0.35-2.49)	p=0.035 OR=1.68 (1.04-2.72)	p=0.73 OR=1.15 (0.52-2.57)	p=0.64 OR=1.16 (0.62-2.17)	p=0.91 OR=1.04 (0.49-2.22)
**RDS**	p=0.003 OR=4.24 (1.63-11.05)	p=0.57 OR=0.74 (0.26-2.11)	p=0.01 OR=1.95 (1.18-3.24)	p=0.8 OR=0.89 (0.36-2.18)	p=0.93 OR=1.03 (0.53-1.99)	p=0.026 OR=2.33 (1.11-4.89)
**FGR**	p=0.3 OR=2.2 (0.5-9.73)	p=0.99 OR=0.988 (0.2-4.95)	p=0.3 OR=1.53 (0.68-3.43)	p=0.86 OR=0.87 (0.2-3.91)	p=0.13 OR=0.39 (0.11-1.34)	p=0.11 OR=2.59 (0.81-8.23)
**pPROM**	p=0.48 OR=1.47 (0.51-4.24)	p=0.87 OR=0.91 (0.29-2.83)	p=0.35 OR=1.3 (0.76-2.24)	p=0.027 OR=2.41 (1.11-5.28)	p=0.83 OR=0.92 (0.45-1.9)	p=0.63 OR=1.23 (0.53-2.86)
**EOI**	p=0.034 OR=3.07 (1.09-8.67)	p=0.15 OR=0.41 (0.12-1.39)	p=0.69 OR=1.13 (0.63-2.04)	p=0.68 OR=1.22 (0.48-3.11)	p=0.41 OR=1.37 (0.66-2.84)	p=0.35 OR=0.62 (0.23-1.68)
**Pneumonia**	p=0.84 OR=1.15 (0.29-4.61)	p=0.99 OR=0.993 (0.23-4.33)	p=0.63 OR=1.2 (0.57-2.51)	p=0.56 OR=0.65 (0.15-2.84)	p=0.15 OR=1.94 (0.8-4.71)	p=0.11 OR=0.28 (0.06-1.32)

No significant differences were observed for low MBL concentrations (<150 ng/ml) between low, medium and high BW groups: 27.7%, 21.1%, and 18.5% (statistical analysis concerning the first and the last mentioned groups is demonstrated in **Table 2**).

It should be mentioned, that when associations of BW with levels of tested collectins in cord sera were analyzed separately in subgroups including neonates born at GA 24-32 weeks and 33-37 weeks, median CL-10 and CL-11 differed significantly in the second mentioned, between babies with GA <1500 g and ≥1500 g [507 ng/ml *vs*, 699 ng/ml, p=0.023 (**Supplementary Figure 5D**) and 253 ng/ml *vs*, 359 ng/ml, p=0.03 (**Supplementary Figure 5E**)]. However, no significant differences were found within shorter GA group (**Supplementary Figures 5A, B**). Furthermore, median MBL concentrations did not differ between babies born with body mass <1500 g and ≥1500 g either at GA 24-32 (**Supplementary Figure 5C**) or 33-37 weeks (**Supplementary Figure 5F**).

No appreciable differences were found for *COLEC11* polymorphisms. C/T or T/T genotypes (rs3820897) had 13 of 41 (31.7%) babies with BW <1500 g, 57/204 (27.9%) of those with BW 1500-2499 g and 55/165 (33.9%) newborns with body mass ≥2500 g. Correspondingly, frequencies of A/G heterozygosity at rs7567833 equalled 3/41 (7.3%), 19/205 (9.3%) and 11/165 (6.7%) (**Table 2**).

The distributions of LXA/O plus O/O *MBL2* genotypes were also similar in the BW groups [BW <1500 g: 5/55 (9.1%); BW 1500-2499 g: 32/278 (11.5%) and BW ≥2500: 24/199 (12.1%) (**Table 2**).

### 3.3 Concentrations of collectins in cord sera from twins

**Figure 4:**
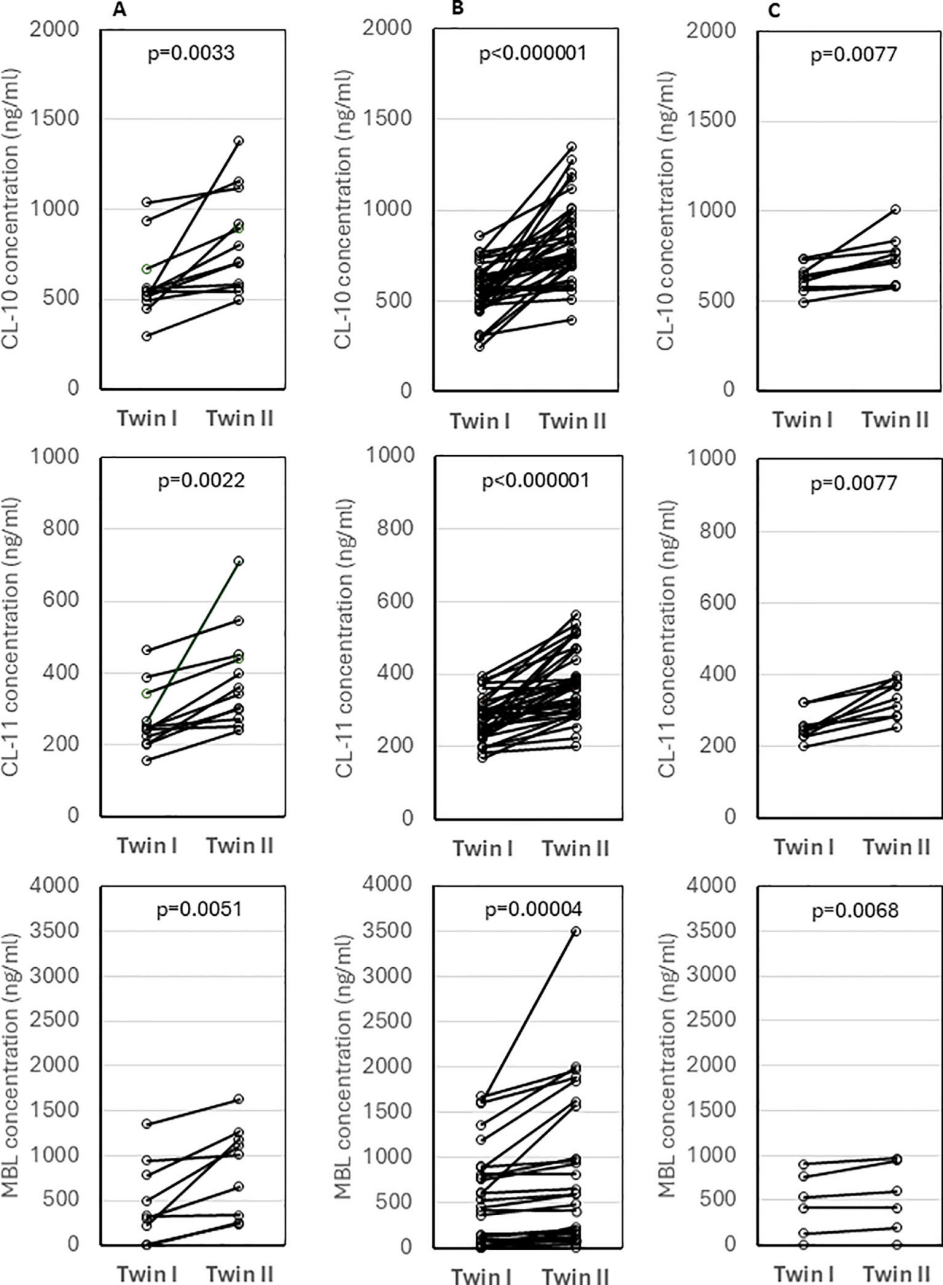
Comparison of concentrations of collectin-10, collectin-11 and mannose-binding lectin between twins of different sex **(A)**, twins of the same sex **(B)** and monochorionic-diamniotic twins **(C)**. Statistical significance values show comparisons with the use of Wilcoxon matched-pairs test.

The concentrations of tested collectins were compared between twins. Despite no differences in birthweight (p>0.05), median levels of CL-10, CL-11 and MBL differed significantly between twins of different sex (data from 12, 12 and 10 pairs, respectively) (**Figure 4A**) and of the same sex (37, 37, 27 pairs, respectively) (**Figure 4B**). The differences were still significant between monochorionic-diamniotic (MCDA) twins (9, 9, and 6 pairs, respectively) (**Figure 4C**).

### 3.4 Associations of collectins with 1’ Apgar score

Median concentrations of both collectin-10 and collectin-11 in cord sera were found to be lower in preterms with 1’ Apgar scores <7 compared with babies scored ≥7 (p<0.05) (**Figures 1C**, **2C**, respectively). However, when Bonferroni correction was applied, the difference for CL-11 occurred insignificant (p>0.0167) (**Figure 2C**). Despite a similar trend, MBL levels did not differ significantly between those groups (**Supplementary Figure 6A**).

When proportions with low concentrations were compared, there were no significant differences: 9/69 (13%) *vs*. 29/329 (8.8%) (CL-10); 9/69 (13%) *vs.* 28/330 (8.5%) (CL-11); 14/68 (20.6%) *vs*. 69/335 (20.6%) (MBL) (more details are presented in **Table 2**).

Thirty-eight/102 (37.3%) and 10/103 (9.7%) babies scored <7 carried at least 1 variant (T) allele at rs3820897 and/or were A/G heterozygotes (rs7567833), respectively, while corresponding frequencies in preterms with higher scores equalled 82/292 (28.7%) and 24/292 (8.7%) (no significant differences, **Table 2**). It should be stressed, however, that multiple logistic regression analysis revealed a significant relationship of rs3820897 with low 1’ Apgar score (OR>1.8) (**Table 3**). No impact of genetically-determined MBL deficiency was found: XA/O or O/O *MBL2* genotypes were carried in 8/78 (10.3%) subjects who scored 1-6 and 41/402 (10.2%) of those who scored 7-10 (**Table 2**).

### 3.5 Associations of collectins with length of hospitalisation and need for intensive care

The median concentrations of both CL-10 and CL-11 in preterm neonates who stayed in hospital for more than two weeks were significantly lower in comparison with those who left the neonatal ward earlier (**Figures 1D**, **2D**, respectively). Furthermore, low (<462 ng/ml) collectin-10 levels were observed more frequently among babies hospitalised for >14 days [19/135 (14.1%) *vs.* 21/280 (7.5%), **Table 2**]. Similarly, a significant difference was observed for collectin-11 [20/135 (14.5%) *vs*. 19/281 (6.8%), **Table 2**].

In contrast, MBL concentrations seemed not to be associated with a need for prolonged hospitalisation (**Supplementary Figure 6B**). Frequency of low MBL (<150 ng/ml) levels did not differ markedly between those groups [31/145 (21.4%) *vs.* 63/293 (21.5%), **Table 2**].

Interestingly, CL-10 (but not CL-11 or MBL) concentrations in cord sera were associated with need-for-stay in the NICU. The median CL-10 level in babies who required intensive care was markedly lower compared with newborns who did not. The difference, however, lost significance when Bonferroni correction was applied (**Supplementary Figure 7A**). No similar relationship was noted in the case of CL-11 (**Supplementary Figure 7B**) or MBL (**Supplementary Figure 7C**). The differences in frequency of low concentrations for CL-10, CL-11, and MBL were not statistically significant: 20/146 (13.7%) *vs*. 20/262 (7.6%), 18/146 (12.3%) *vs*. 21/263 (8%), 35/160 (21.9%) *vs.* 60/284 (21.1%), respectively (**Table 2**).

When data from preterms who required prolonged (>4 days) intensive care were compared with results from newborns who spent 0-4 days in NICU (all survivors), a similar association of CL-10 was found (**Figure 1E**). Furthermore, low CL-10 concentrations were more common among patients requiring prolonged NICU stay when compared with the reference group [15/97 (15.5%) *vs.* 25/311 (8%), **Table 2**]. That association has moreover been confirmed by multiple logistic regression analysis (**Table 3**). However, no similar relationships were found with CL-11 (**Supplementary Figure 8A**) or MBL (**Supplementary Figure 6C**). None of the genetic polymorphisms investigated were associated with length of hospitalisation or with stay in NICU The frequencies of *COLEC11* C/T or T/T genotypes, A/G heterozygosity and MBL deficiency-associated genotypes were 47/136 (34.6%), 12/136 (8.8%) and 20/171 (11.7%) when preterms were hospitalised for more than 2 weeks and 77/270 (28.5%, p=0.25), 22/271 (8.1%, p=0.85) and 38/351 (10.8%, p=0.77), when their stay in hospital was shorter (**Table 2**).

Among babies who underwent intensive care, 52/143 (36.4%) carried C/T or T/T genotypes (*COLEC11* rs3820897), 15/143 (10.5%) – A/G genotype (rs7567833) and 22/190 (11.6%) had MBL-deficient genotypes. Corresponding frequencies in newborns who did not stay in NICU equalled 73/261 (28%, p=0.092), 19/262 (7.3%, p=0.266) and 36/341 (10.6%, p=0.72). No significant differences in distribution of studied genotypes were observed in regard to prolonged intensive therapy: 33/94 (35.1%) *vs.* 92/310 (39.7%), p=0.37 (*COLEC11* C/T or T/T); 9/94 (9.6%) *vs.* 25/311 (8%), p=0.67 (*COLEC11* A/G); 18/130 (13.8%) *vs.* 40/399 (10%), p=0.23 (*MBL2* XA/O or O/O) (**Table 2**).

### 3.6 Associations of collectins with need for respiratory support

Cord serum levels of CL-10 were significantly lower in preterm newborns requiring respiratory support compared with spontaneously breathing neonates (**Figure 1F**). A similar, but statistically non-significant, trend was observed for CL-11 (**Supplementary Figure 8B**) and MBL (**Supplementary Figure 6D**).

Low concentrations of CL-10 were more common among babies who needed respiratory support compared with those who did not [21/122 (17.2%) *vs.* 20/294 (6.8%), **Table 2**]. This relationship was confirmed by multiple logistic regression analysis (OR>3.7) (**Table 3**). A similar association was found for CL-11 [19/122 (15.6%) *vs.* 21/295 (7.1%)] but not MBL [30/128 (23.4%) *vs.* 64/318 (20.1%)] (**Table 2**). Consequently, neonates with low CL-10 or CL-11 needed longer respiratory support [mean 4.7 days *vs.* 2.2 days for low CL-10 (p=0.0059) and 4.3 days *vs.* 2.3 days for low CL-11 (p=0.026)]. Low MBL concentration in cord serum had no impact on the length of respiratory support (2.8 days *vs.* 2.3 days, p=0.22).

The *COLEC11* rs3820897 SNP could be weakly associated with the need for respiratory support: the C/T or T/T genotypes were found more frequently in the affected, compared with the reference group [45/117 (38.5%) *vs.* 83/294 (28.2%), **Table 2**]. Again, this finding was confirmed in multiple logistic regression (OR=1.69, **Table 3**). No such relationship was found in the case of *COLEC11* rs7567833 heterozygosity [10/118 (8.5%) *vs.* 24/294 (8.2%)] or MBL primary deficiency [17/156 (10.9%) *vs.* 44/376 (11.7%)] (**Table 2**).

### 3.7 Associations of collectins with respiratory distress syndrome

Preterm newborns who suffered from RDS had significantly lower median concentrations of CL-10, CL-11 and MBL in cord sera than babies without that complication. Those associations remained significant after correction for multiple comparisons. The individual values, depending on RDS incidence are presented in **Figure 1G** (CL10), **Figure 2E** (CL-11) and **Figure 3C** (MBL). Intriguingly, median CL-10 and CL-11, differed significantly in babies born at GA ≥33 weeks but not in those born at GA <33 weeks (**Supplementary Figures 7D, E**). An opposite relationship was found for MBL (**Supplementary Figure 6F**). Corresponding associations were observed depending on birthweight: RDS patients with BW ≥1500 g had lower levels of both CL-10 and CL-11 compared with unaffected babies (**Supplementary Figures 7G, H**) while MBL concentration differed in the other (BW<1500 g) group (**Supplementary Figure 7I**).

Low CL-10 concentrations were observed more frequently among neonates with than without RDS [17/104 (16.3%) *vs.* 23/313 (7.3%), **Table 2**] and multiple regression analysis confirmed low CL-10 to be a risk factor for disease development (OR>4.2) (**Table 3**). Similar, but statistically non-significant, trends were found for CL-11 [15/104 (14.4%) *vs.* 24/314 (7.6%)] and MBL [29/107 (27.1%) *vs.* 64/338 (18.9%) (**Table 2**).

Interestingly, as many as 41/101 (40.6%) preterm newborns who developed RDS were carriers of the variant T allele at *COLEC11* rs3820897 polymorphism, compared with 87/311 (29%) of those who did not (significant difference, see **Table 2**). That association was confirmed in multiple logistic regression analysis (OR=1.69, **Table 3**), but G allele carriers at rs7567833 did not differ significantly in that regard [7/101 (6.9%) *vs.* 27/311 (8.7%), **Table 2**]. MBL deficiency-associated genotypes were commoner among babies who developed RDS [21/131 (16%) *vs.* 40/401 (10%), **Table 2**]. That relationship reached statistical significance after multiple analysis (OR=2.3, **Table 3**). Conversely, A/A genotypes were significantly less common in RDS compared with the reference group [57.3% *vs.* 68.3%, OR=0.62, 95 CI (0.41-0.93), p=0.021].

### 3.8 Associations of collectins with fetal growth restriction

The median concentration of collectin-10 in cord sera from preterm neonates diagnosed with fetal growth restriction was significantly lower than in eutrophic newborns (**Figure 1H**). However, the difference lost statistical significance when correction for multiple comparisons was applied (p>0.0167). Although a similar trend was observed in the case of CL-11, the difference was not statistically significant (**Supplementary Figure 8C**). Median MBL was slightly higher in the grow restriction group, in comparison with the reference group (**Supplementary Figure 6E**).

No significant differences were found when the distribution of low serum levels was compared with regard to FGR: 5/32 (15.6%) *vs.* 36/369 (9.8%) (CL-10); 4/32 (12.5%) *vs.* 35/370 (9.5%) (CL-11); 10/47 (21.3%) *vs.* 85/399 (21.3%) (MBL) (**Table 2**).

Similarly, genotype frequencies of both *COLEC11* or *MBL2* did not differ significantly between preterms diagnosed with FGR and those with no FGR: 13/32 (40.6%) *vs.* 111/364 (30.5%), (C/T or T/T, *COLEC11* rs3820897); 3/32 (9.4%) *vs.* 28/365 (7.7%) (A/G, *COLEC11* rs7567833); 9/62 (14.5%) *vs.* 51/470 (10.9%) (XA/O or O/O, *MBL2*) (**Table 2**).

### 3.9 Associations of collectins with preterm premature rupture of membranes

The median concentrations of CL-10, CL-11 and MBL were similar in babies from pregnancies with and without premature rupture of membranes (**Supplementary Figures 7J–L**, respectively). Also, the proportions of low serum values for all three collectins did not differ appreciably between the groups: 9/79 (11.4%) *vs.* 32/339 (9.4%) (CL-10); 8/79 (10.1%) *vs.* 32/340 (9.4%) (CL-11); 19/88 (21.6%) *vs.* 75/358 (20.9%) (MBL) (**Table 2**).

However, heterozygosity for *COLEC11* rs7567833 was carried more frequently by babies born with pPROM compared with newborns without [11/77 (14.3%) *vs.* 23/337 (6.8%), **Table 2**]. That association was confirmed in multiple logistic regression analysis (OR>2.4, **Table 3**). No such relationships were found for the other collectin genotypes investigated [26/77 (33.8%) *vs.* 102/336 (30.4%), (*COLEC11* rs3820897); 12/101 (11.9%) *vs.* 49/432 (11.3%) (*MBL2* XA/O or O/O)] (**Table 2**).

### 3.10 Associations of collectins with early-onset infections

The concentrations of CL-10, CL-11 and MBL seemed not to be associated with susceptibility to early-onset perinatal infections (**Supplementary Figures 7M–O**, respectively). No appreciable differences were noted in frequencies of low concentrations of tested proteins, although a trend was apparent for CL-10 [10/66 (15.2%) *vs.* 31/351 (8.8%), **Table 2**]. Moreover multiple regression analysis confirmed that association (OR>3) (**Table 3**). Corresponding frequencies for CL-11 [6/66 (9.1%) *vs.* 34/352 (9.7%)] or MBL 16/67 (23.9%) *vs.* 70/348 (20.1%)] occurred virtually the same (**Table 2**).

No significant differences were observed in the distribution of genotypes investigated [21/65 (32.3%) *vs.* 106/347 (30.5%) (C/T or T/T, *COLEC11* rs3820897); 7/65 (10.8%) *vs.* 27/348 (7.8%) (A/G, *COLEC11* rs7567833); 6/76 (7.9%) *vs.* 48/421 (11.4%) (XA/O or O/O, *MBL2*)] (**Table 2**).

Additionally, neither concentrations of collectins nor frequencies of variant alleles of corresponding genes differed significantly between preterms with pneumonia and those without (**Table 2**).

### 3.11 Expression of collectins in placenta

The expression of *COLEC10*, *COLEC11* and *MBL2* genes was analysed in 35 (GA 30-41, mean 36.5) placenta samples from live births. *MBL2* mRNA was found in 27, while the others tested genes were expressed at detectable levels in all placentas. The expression of *COLEC10* and *COLEC11* (but not *MBL2*) was significantly higher in the maternal, in comparison with the fetal part of the placenta. Detailed data are presented in **Table 4**. Significant correlations were observed between mRNA levels in maternal and fetal parts [r=0.44, p=0.0091 (**Supplementary Figure 9A**), r=0.483, p=0.0038 (**Supplementary Figure 9B**) and r=0.676, p=0.00008 (**Supplementary Figure 9C**), respectively].

**Table 4:** The comparison of expression of mRNA from the *COLEC10, COLEC11* and *MBL2* genes in maternal and fetal parts of placentas from live births.

Gene	mRNA expression level (median 2^-ΔCt^)		Statistical significance
	maternal part	fetal part	
*COLEC10*	1.232 x 10^-2^	6.39 x 10^-3^	p=0.005
*COLEC11*	1.253 x 10^-3^	5.265 x 10^-4^	p<0.0001
*MBL2*	7.533 x 10^-3^	1.318 x 10^-3^	p>0.05

## 4 Discussion

Both CL-10 and CL-11 are thought to be important for fetal development as mutations of *COLEC10* and *COLEC11* genes have been reported in patients diagnosed with 3MC syndrome [reviewed in (10)]. However, no data have been published previously concerning possible relationships of those collectins with spontaneous preterm births and their associated complications. Much more data are available about MBL in neonatal health and disease [reviewed in (5, 6)].

The well-established relationship between *MBL2* genotype and MBL concentration (21) was confirmed. We found no such relationship concerning the *COLEC11* rs3820897 (promoter, -9570 C>T) SNP, previously suggested to influence the serum level of CL-11 (16). The difference might reflect age-dependent mechanisms of regulation of gene expression, including the effect of sex hormones. We also found no influence on the serum concentration of another polymorphism (rs7567833, +39618 A>G, His219Arg), and in that regard, our two studies are in agreement (16). Although PolyPhen-2 tool (http://genetics.bwh.harvard.edu/pph2/) classified that SNP as “benign”, it cannot be excluded that amino acid exchange affects CL-11 binding affinity (16). Despite no impact of tested polymorphisms on collectin-11 levels, their clinical significance cannot be excluded: the minor alleles at rs7567833 and rs3820897 were more frequent among pPROM cases and among preterms requiring respiratory support, respectively.

Intriguingly, the median concentration of CL-11 in cord sera from preterm neonates was exactly the same as in sera from healthy adults (0.32 µg/ml) while CL-10 levels differed markedly: 0.63 µg/ml reported here for babies and 1.87 µg/ml for blood donors (16), despite highly significant CL-10/CL-11 correlation in both studies (r=0.91 and r=0.741, respectively). That may suggest age-dependent differences in *COLEC10* gene expression. Such differences have previously been reported for other complement-activating lectins as MBL, ficolins as well as for associated serine protease MASP-2 (23, 28). Vinuela et al. identified as many as 137 genes expressed differentially with age (independently of environmental factors) what, among others, may reflect different genetic regulation or mRNA splicing (24). As mentioned, *COLEC10* rs149331285 and rs148350292 were not submitted for statistical analysis due to the lack or low frequency of variant alleles, respectively. Based on data from 296 Danish blood donors, their corresponding MAF were previously reported as 0.007 (rs149331285) and 0.005 (rs148350292) (16). Here, we analyzed a larger, specific group (preterm neonates) from another (Polish) population which might be considered a reason for the differences noted (MAF<0.001 and MAF=0.002, respectively).

It has long been known that twins may have dramatically different cord MBL concentrations (25), as illustrated in **Figure 4**. In dizygotic siblings, this is obviously due to the inheritance of different *MBL2* genotypes. This could be considered evidence that cord MBL is derived exclusively from the fetus and not from the maternal circulation. However, marked differences in MBL, CL-10 and CL-11 concentrations in monochorionic-diamniotic twin pairs were also noted, therefore, epigenetic mechanisms of regulation of their expression cannot be excluded. Even in monochorionic pregnancies, the umbilical cords may, for example, differ, to some extent, in maternal blood flow rate what may in turn affect transportation of nutrients, hormones, *etc.* It should be however stressed that Husby et al. (26) reported highly significant correlation between MBL levels in both monozygotic and dizygotic twins of the same sex, aged 6-9 years (r=0.97, p<0.01 and r=0.22, p<0.01, respectively). They suggested the importance of non-additive genetic and non-shared environmental factors, and estimated heritability to equal 0.96 [95% CI (0.92-0.97)] (26).

Despite significant correlations of CL-10, CL-11 and MBL concentrations with gestational age, only CL-10 differed significantly between preterms with shorter (<33 weeks) and longer GA (33-37 weeks). On the other hand, low CL-11 concentrations were almost as common as low CL-10 amongst babies born at GA ≤32. The majority of, but not all, earlier studies concerning MBL demonstrated a significant correlation between MBL levels and gestational age and/or lower average concentrations in preterm compared with term neonates (22, 27–31). In contrast, in our previous investigation, neither significant correlation of MBL with GA nor a greater difference in median MBL concentration in cord sera between term and preterm newborns was noted (21). Probably, reported here and elsewhere, the correlations reflect rather differences in ability of fetal liver to produce collectins and release them into the blood, depending on gestational age than direct cause-and-effect relationship between low protein concentration and shortened GA. However, the median CL-10 concentration in cord sera was found to be relatively low within the group of preterms affected by fetal growth restriction.

Here, we found no impact of *COLEC10, COLEC11* or *MBL2* polymorphisms on gestational age. Only *MBL2* has been previously studied in that respect. Some papers reported differences in allele distribution between preterm and term newborns (21, 32, 33), while others indicated no such relationship (30, 34). Our data presented here, concerning preterms only, seem to confirm those published by Frakking et al. (30) and Grumach et al. (34).

For the first time, we found significant relationships between concentrations of CL-10 and CL-11 in cord sera and birthweight (**Figures 1B**, **2B**), but not with *COLEC10* or *COLEC11* polymorphisms. That, at least to some extent, may reflect differences in size of liver, being the main site of synthesis of investigated collectins. However, MBL concentration did not correlate with birthweight and this adds to an inconsistent literature. For example, Hartz et al. (35) found no association of MBL with very low birthweight (VLBW), while Cakmak et al. (36) found the opposite.

We report here associations of CL-10 and, to a lesser extent, CL-11 with a poor Apgar score and an extended hospital stay. There is no proof that those relationships are causal, but they could be an indication of an important role in embryogenesis and/or early ontogenesis.

A major finding of this study has been the clear association of CL-10, CL-11 and MBL with respiratory distress syndrome (RDS). RDS is considered to be a leading aetiology of neonatal respiratory failure (NRF) and the main cause of NRF-related deaths (37). It is a multifaceted and intricate disease that typically manifests in newborns with lung underdevelopment and deficiency of lung surfactant. It is closely associated with the onset of bronchopulmonary dysplasia (BPD) and numerous other adverse events (38), and may even lead to respiratory problems in adulthood. Ours is the first study to relate CL-10 and CL-11 to RDS, but MBL has previously been investigated by others (39, 40). Since we previously also found low concentrations of ficolin-2 in RDS patients (from the same cohort), it may be supposed that impaired activation of complement *via* the lectin pathway contributes to the development of this complication. On the other hand, we cannot exclude the consumption of collectins/ficolins due to the involvement of complement in RDS pathogenesis or (considering the relative high frequency of primary MBL deficiency in patients), both mentioned mechanisms. The different role of MBL compared with CL-10 or CL-11 might be assumed based on different GA and BW ranges in which their significance is observed. Lower concentrations of intact C4 and higher - of complement activation products in neonatal RDS were reported and proposed to associated with poor response to surfactant treatment (41–43).

Numerous studies have addressed *MBL2* genotypes and/or serum MBL concentrations in relation to perinatal infections, including sepsis. Diversely designed studies of various cohorts/populations often led to contradictory conclusions: some suggested MBL deficiency was a risk factor, while others found no role for that collectin [reviewed in (5, 6)]. We found no association between *MBL2* polymorphisms or MBL in cord sera and early onset infections (including several cases of sepsis) in preterms. The multicentre studies, with the use of an unified method for measurement of MBL concentration and determination of the same set of polymorphisms might address the mentioned inconsistencies and lead to *consensus*. However, differing frequencies of variant alleles for commonly tested *MBL2* polymorphisms in various ethnic groups may complicate it. Although a possible association with low CL-10 concentration was noted, we found little evidence for an anti-microbial role for those collectins before birth. On the other hand, low level of CL-10 seemed to predict the need for prolonged hospitalisation (including NICU), not necessarily associated with infection, and the need for respiratory support. That may suggest its particularly important role in keeping neonatal homeostasis. Although, in general, complement-activating collectins studied here are closely related both structurally and functionally, they may play distinct roles (and therefore different clinical associations) due to their different affinity, hepatic and extra-hepatic expression, ability (or its lack) to form heterocomplexes, *etc.*

The complement system is thought to be important for placental and fetal development from implantation (44). Here, we have reported expression of *COLEC10*, *COLEC11* and *MBL2* genes at the mRNA level in both preterm and term placentas. This confirms the work of others (45–47) who described the expression of *COLEC10* and *COLEC11* genes as well as corresponding proteins in full-term placentas. However, for the first time, we reported significant differences between expression levels of the mentioned genes in maternal and fetal parts. At least in the case of *COLEC10* gene that might be a reflection of generally higher expression in adults compared with neonates (*vide* difference in median concentration of CL-10 in the sera, not noted for CL-11). According to data from Genotype-Tissue Expression (GTEx) Portal (www.gtexportal.org), *COLEC11* expression (in contrast to *COLEC10*) is relatively high in the ovary (but, on the other hand, low in the uterus in both cases). Verification of the hypothesis whether the mentioned phenomena explain our findings needs however further investigation. Our data are moreover consistent with the observations of Kilpatrick et al. (48) that MBL protein can be detected in first-trimester placenta and Yadav et al. (49), who found *MBL2* transcript in term placentas. The presence of *COLEC10* and *COLEC11* (in contrast to *MBL2*) transcripts in all tested samples seems to confirm particular importance of collectin-10 and colectin-11 during embryogenesis.

## 5 Conclusions

Both CL-10 and CL-11 may be important for human embryo-/ontogenesis, based on associations of their gene mutations with 3MC syndrome. We have found for the first time a relationship between low concentrations of CL-10 in cord sera and very premature births at GA ≤32 weeks. Both CL-10 and CL-11 were found to be associated with very low birthweight, low Apgar 1’ score, and prolonged hospitalisation. Lower median CL-10 was associated with fetal growth restriction, while *COLEC11* heterozygosity for a polymorphism affecting CL-11 activity was more common in pPROM, compared with the corresponding reference groups. Both CL-10 and CL-11 were found to be associated with RDS, and CL-10 may influence susceptibility to early-onset infections. MBL was also associated with RDS, but did not share the other clinical correlates of CL-10/CL-11. These complement-activating collectins [as well as ficolin-2 (12–14)] may be important for keeping homeostasis in preterms. The mentioned numerous associations have, however, to be confirmed in an independent study to verify the usefulness of those innate immunity factors as prognostic/diagnostic markers, possibly in a common panel.

## Data Availability

The original contributions presented in the study are included in the article/**Supplementary Material**. Further inquiries can be directed to the corresponding author.
