# Supplementary material for: Clinical associations of complement-activating collectins, collectin-10, collectin-11 and mannose-binding lectin in preterm neonates
Source: Front Immunol. 2024 Oct 11;15:1463651. doi: 10.3389/fimmu.2024.1463651 (PMC11502412; doi:10.3389/fimmu.2024.1463651)
Supplement: Supplementary file 1 [file DataSheet1.pdf]

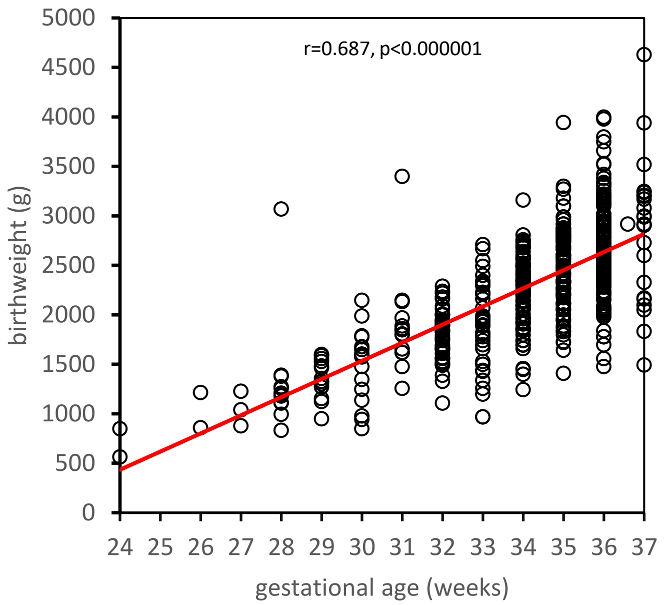

Supplementary Figure 1. Correlation (Spearman) between gestational age and birthweight. Trend line is shown in red.

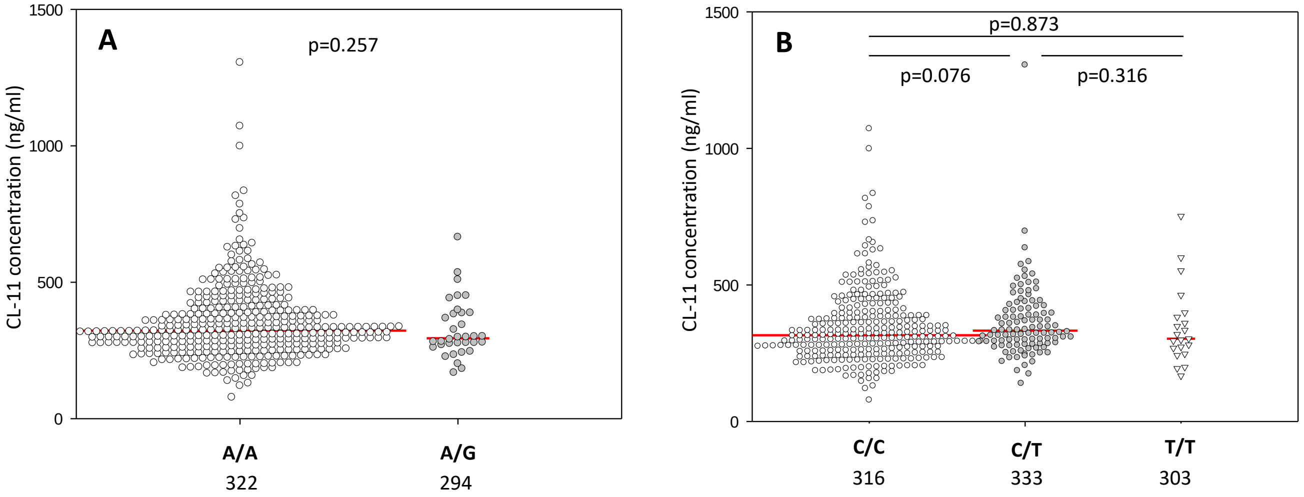

Supplementary Figure 2. Individual concentrations of collectin-11 in cord sera from preterm neonates, depending on *COLEC11* gene polymorphisms: rs7567833 (exon 7, +39618 A>G, His219Arg) (**A**) and rs3820897 (promoter region, -9570 C>T) (**B**). Red bars represent median values (demonstrated as numbers below the graphs). Statistical significance values show comparisons with the use of Mann-Whitney *U* test.

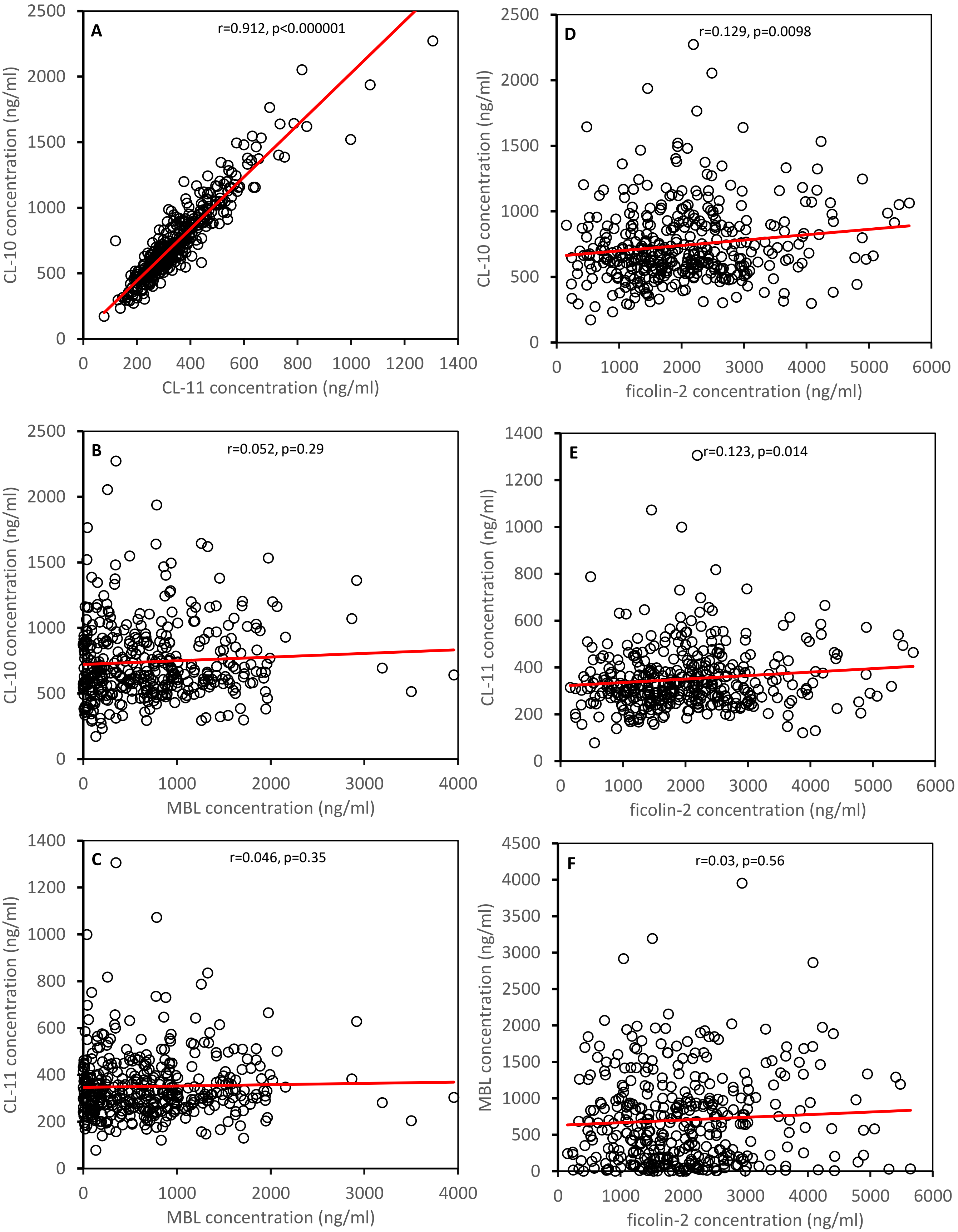

Supplementary Figure 3. Correlations (Spearman) between concentrations of collectin-10 and collectin-11 (**A**), collectin-10 and mannose-binding lectin (**B**), collectin-11 and mannose-binding lectin (**C**), collectin-10 and ficolin-2 (**D**), collectin-11 and ficolin-2 (**E**), and mannose-binding lectin and ficolin-2 (**F**). Trend lines are shown in red.

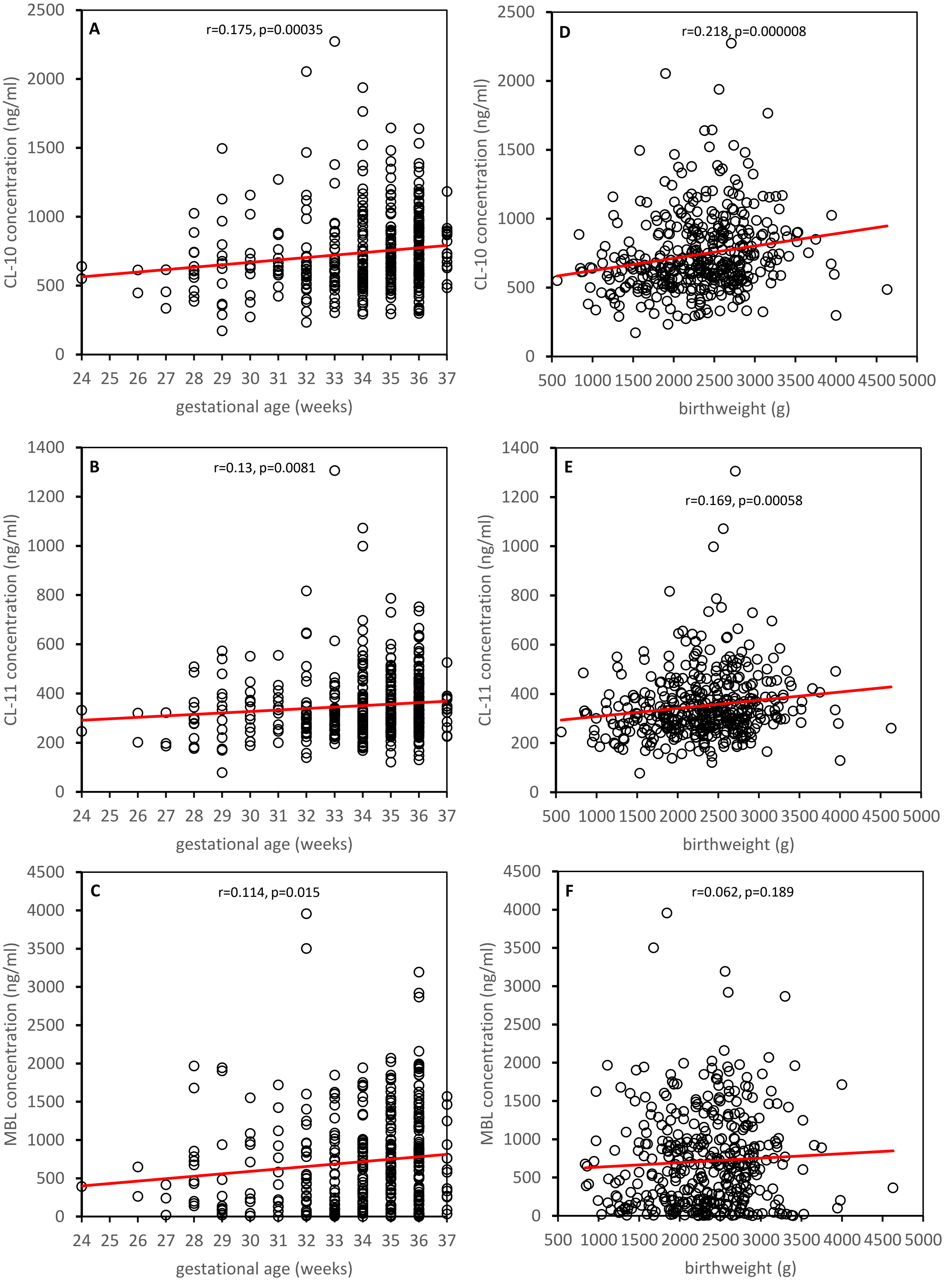

Supplementary Figure 4. Correlations (Spearman) of gestational age with concentrations of collectin-10 (**A**), collectin-11 (**B**) and mannose-binding lectin (**C**); Correlations (Spearman) of birthweight with concentrations of collectin-10 (**D**), collectin-11 (**E**) and mannose-binding lectin (**F**). Trend lines are shown in red.

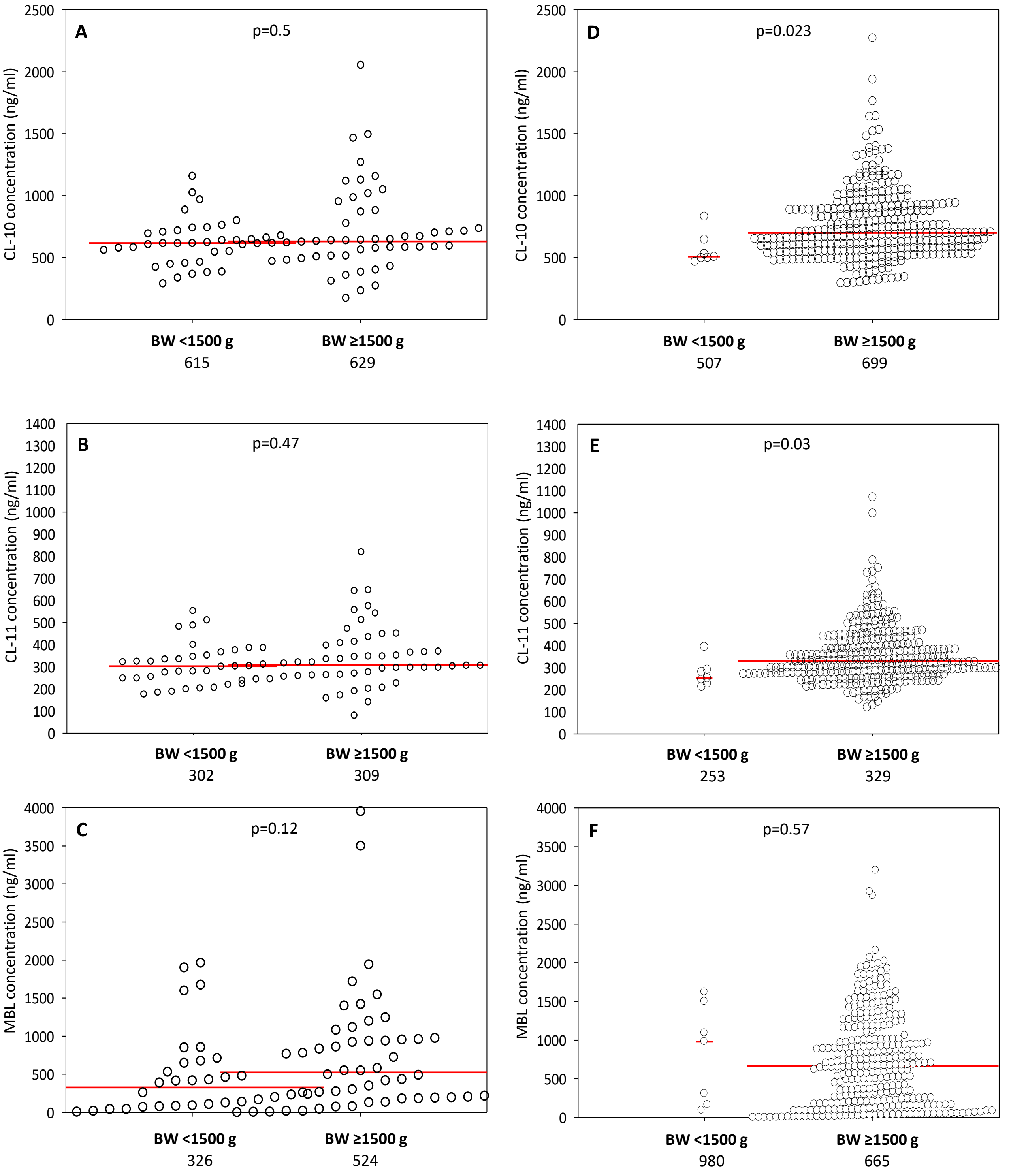

Supplementary Figure 5. Individual concentrations of collectin-10 (**A, D**), collectin-11 (**B, E**) and mannose-binding lectin (**C, F**) in preterm neonates born at gestational age 24-32 weeks (**A-C**) and 33-37 weeks (**D-F**), depending on birthweight. Red bars represent median values (demonstrated as numbers below the graphs). Statistical significance values show comparisons with the use of Mann-Whitney *U* test.

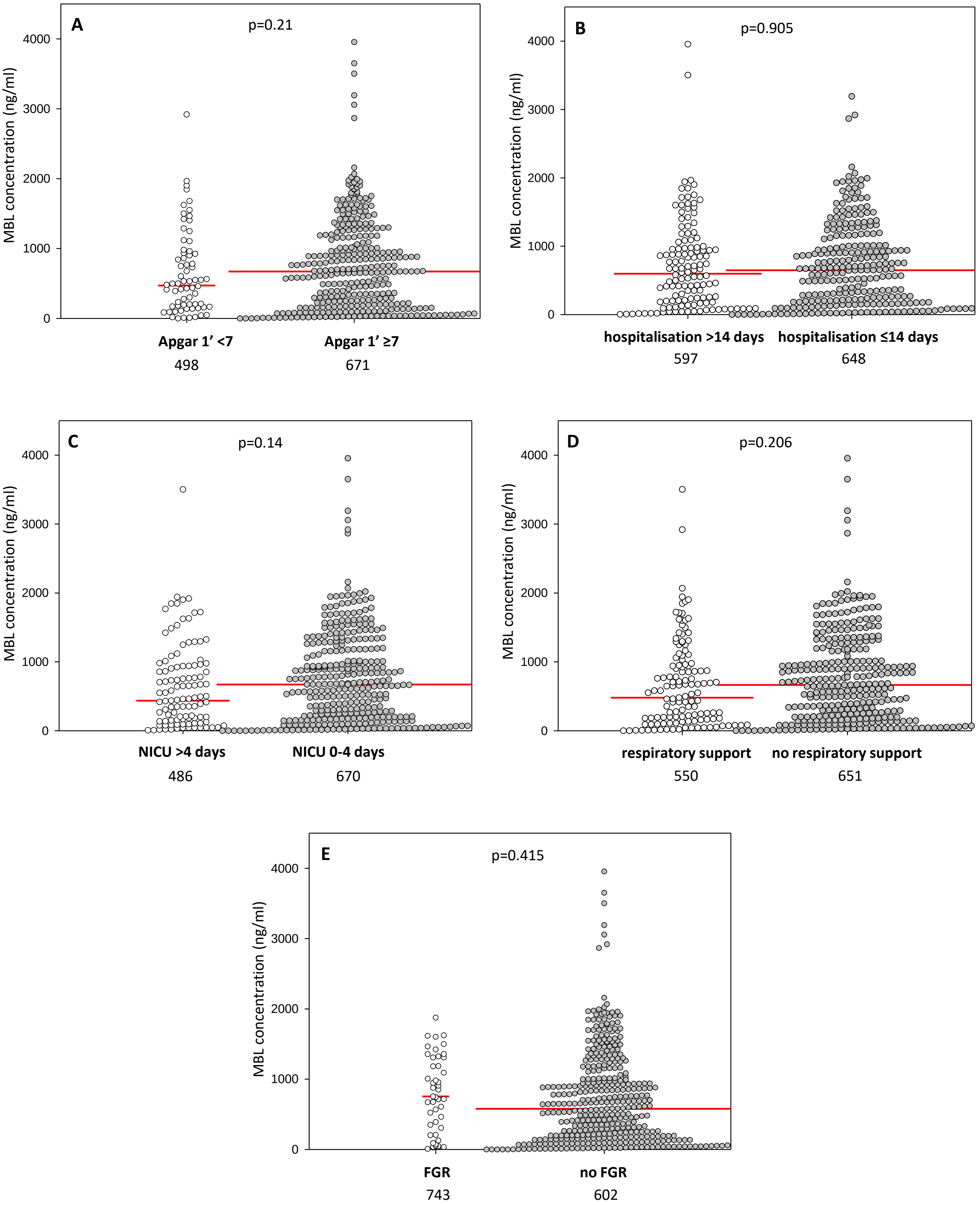

Supplementary Figure 6. Individual concentrations of mannose-binding lectin in preterm neonates, depending on 1’ Apgar score (**A**), length of hospitalisation (**B**), length of intensive care (**C**), need for respiratory support (**D**), and incidence of fetal growth restriction (**E**). Red bars represent median values (demonstrated as numbers below the graphs). Statistical significance values show comparisons with the use of Mann-Whitney *U* test.

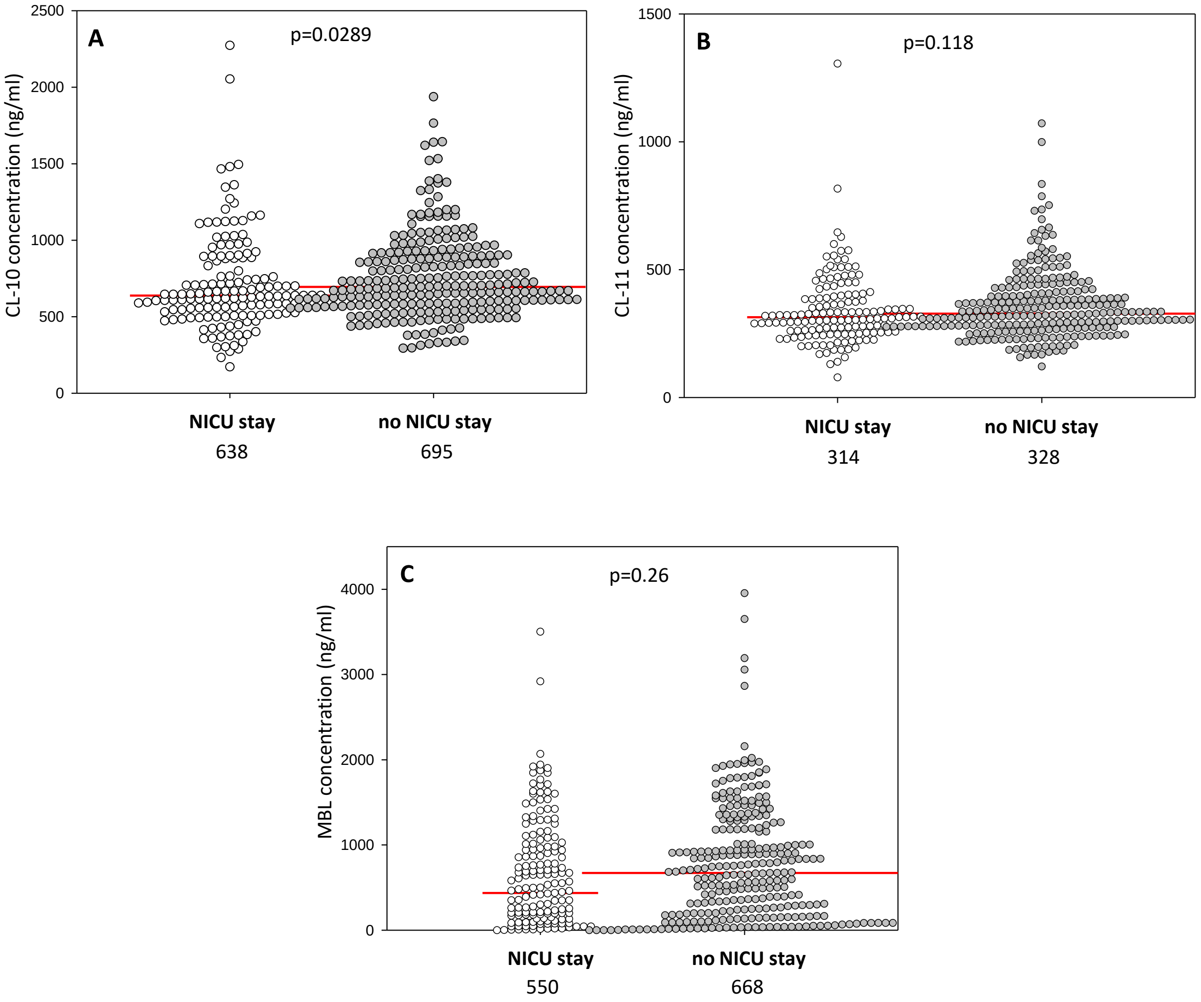
A
p=0.0289
CL-10 concentration (ng/ml)
2500
2000
1500
1000
500
0
NICU stay
638
no NICU stay
695
B
p=0.118
CL-11 concentration (ng/ml)
1500
1000
500
0
NICU stay
314
no NICU stay
328
C
p=0.26
MBL concentration (ng/ml)
4000
3000
2000
1000
0
NICU stay
550
no NICU stay
668

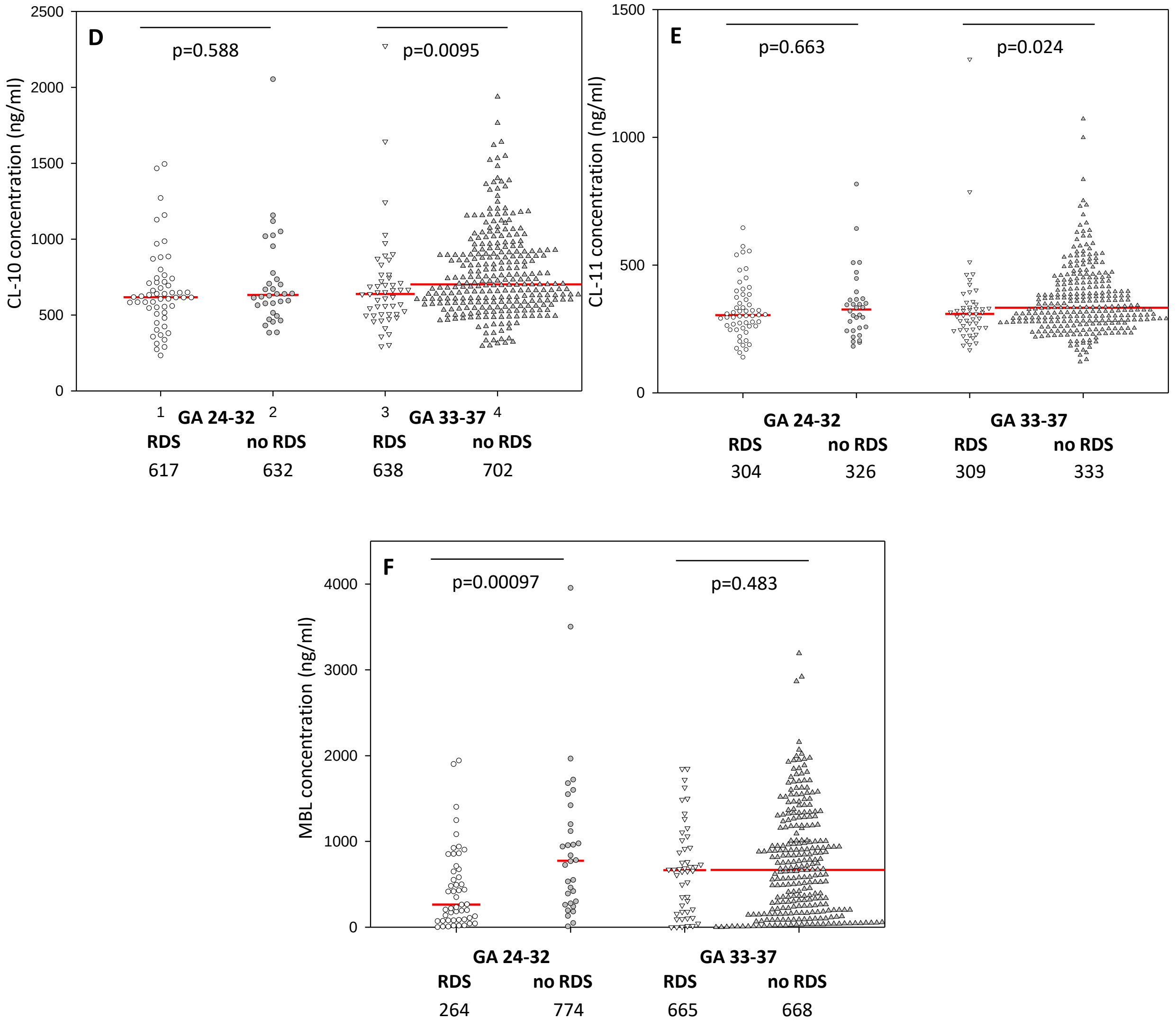
D
p=0.588
p=0.0095
CL-10 concentration (ng/ml)
2500
2000
1500
1000
500
0
1
2
3
4
GA 24-32
GA 33-37
RDS
no RDS
RDS
no RDS
617
632
638
702
E
p=0.663
p=0.024
CL-11 concentration (ng/ml)
1500
1000
500
0
GA 24-32
GA 33-37
RDS
no RDS
RDS
no RDS
304
326
309
333
F
p=0.00097
p=0.483
MBL concentration (ng/ml)
4000
3000
2000
1000
0
GA 24-32
GA 33-37
RDS
no RDS
RDS
no RDS
264
774
665
668

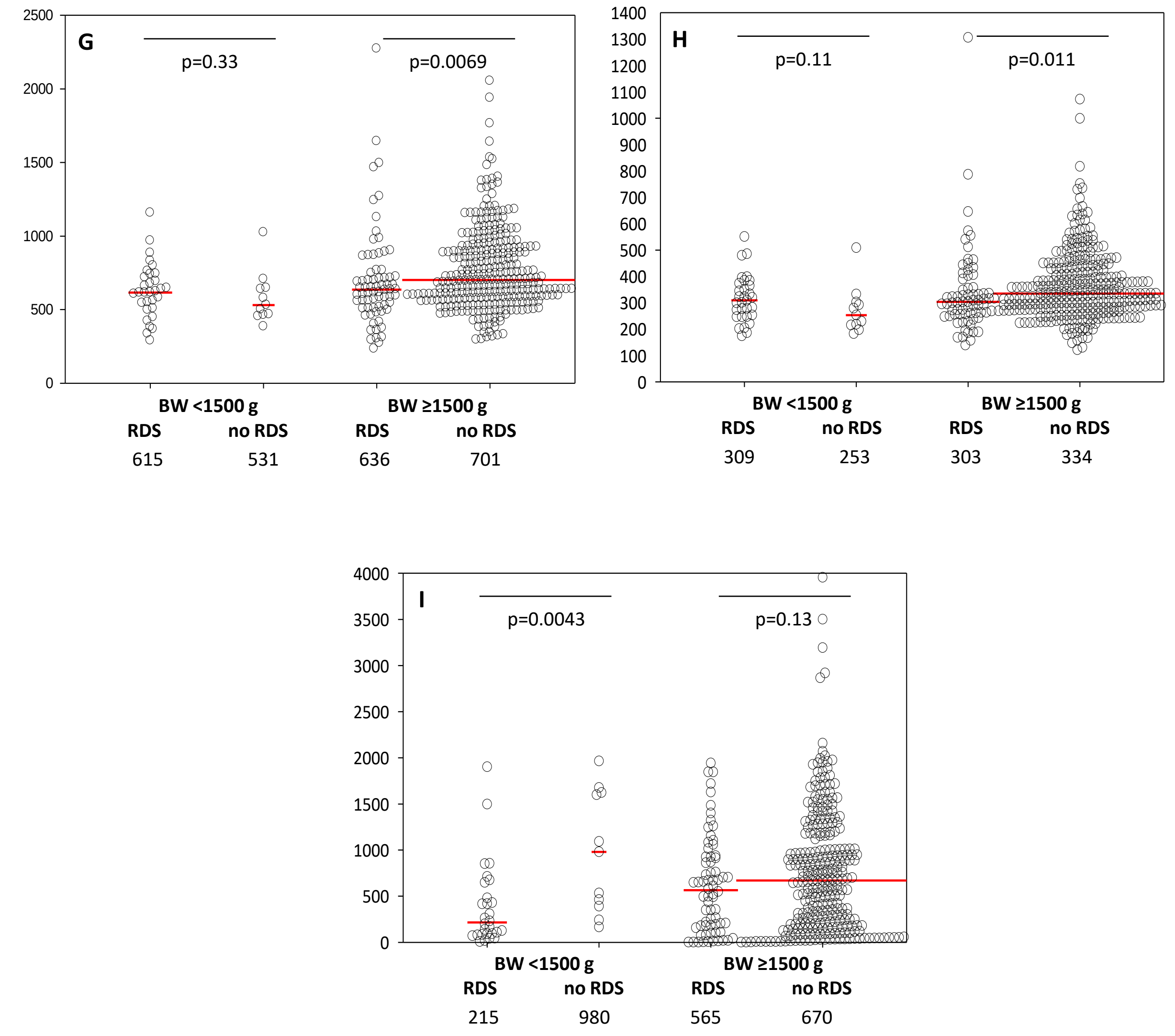
G
p=0.33
p=0.0069
2500
2000
1500
1000
500
0
BW <1500 g
BW ≥1500 g
RDS
no RDS
RDS
no RDS
615
531
636
701
H
p=0.11
p=0.011
1400
1300
1200
1100
1000
900
800
700
600
500
400
300
200
100
0
BW <1500 g
BW ≥1500 g
RDS
no RDS
RDS
no RDS
309
253
303
334
I
p=0.0043
p=0.13
4000
3500
3000
2500
2000
1500
1000
500
0
BW <1500 g
BW ≥1500 g
RDS
no RDS
RDS
no RDS
215
980
565
670

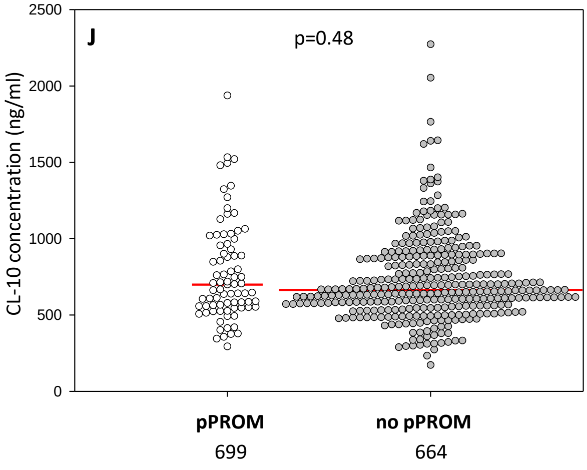
J
p=0.48
CL-10 concentration (ng/ml)
2500
2000
1500
1000
500
0
pPROM
699
no pPROM
664

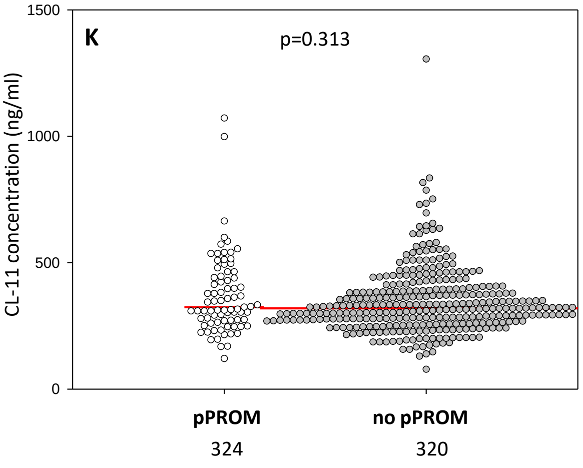
K
p=0.313
CL-11 concentration (ng/ml)
1500
1000
500
0
pPROM
324
no pPROM
320

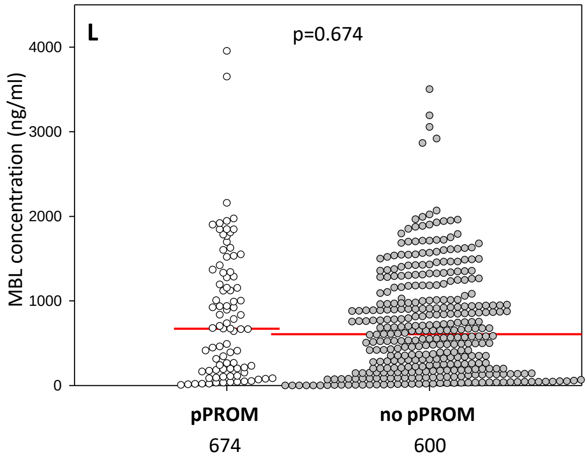
L
p=0.674
MBL concentration (ng/ml)
4000
3000
2000
1000
0
pPROM
674
no pPROM
600

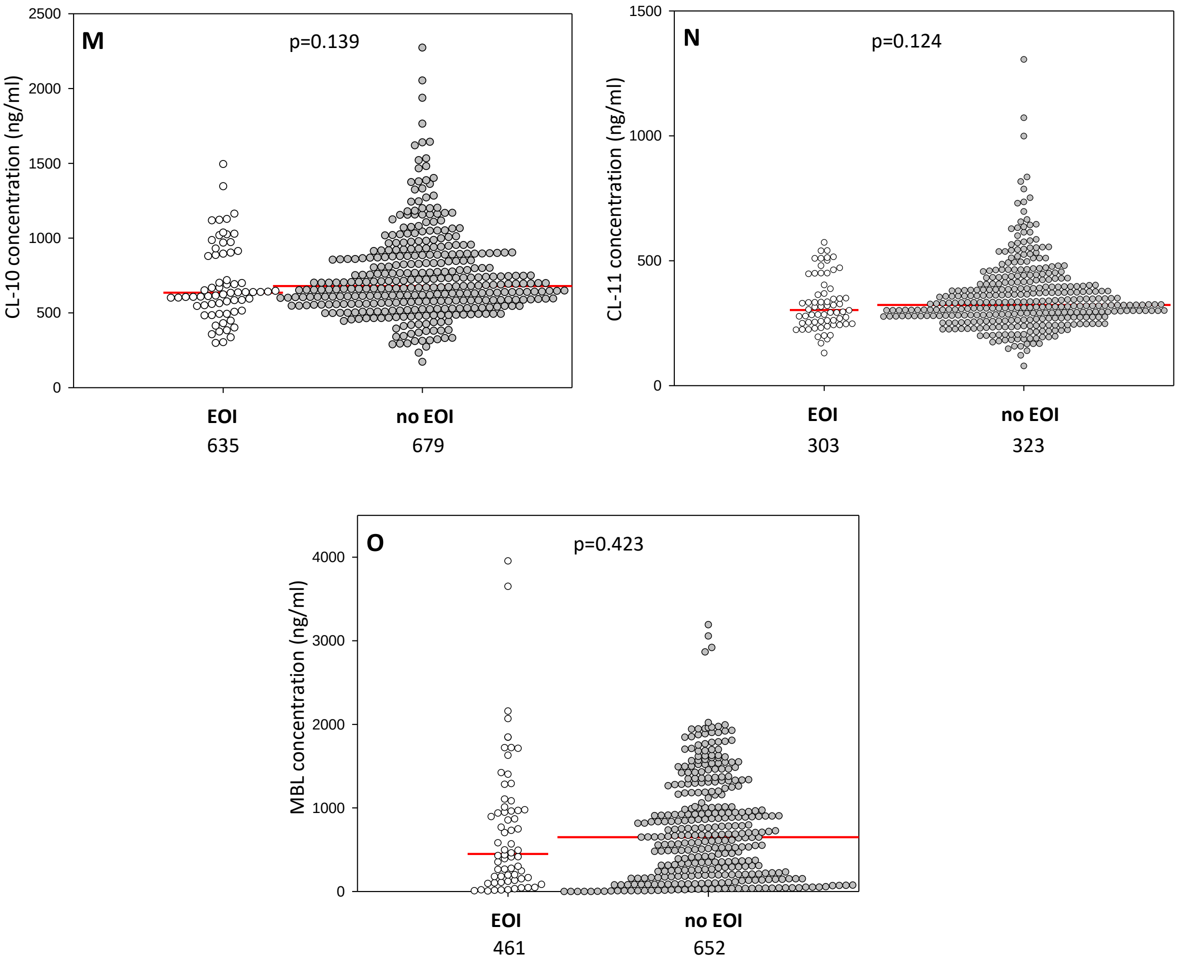

Supplementary Figure 7. Individual concentrations of collectin-10 (**A, D, G, J, M**), collectin-11 (**B, E, H, K, N**) and mannose-binding lectin (**C, F, I, L, O**) in preterm neonates who required intensive care and who were not staying in neonatal intensive care unit (**A-C**); diagnosed with respiratory distress syndrome (RDS) and free of RDS, depending on gestational age (**D-F**); diagnosed with respiratory distress syndrome (RDS) and free of RDS, depending on birthweight (**G-I**); depending on incidence of preterm premature rupture of membranes (**J-L)**, and early onset infections (**J-L**). Red bars represent median values (demonstrated as numbers below the graphs). Statistical significance values show comparisons with the use of Mann-Whitney *U* test.

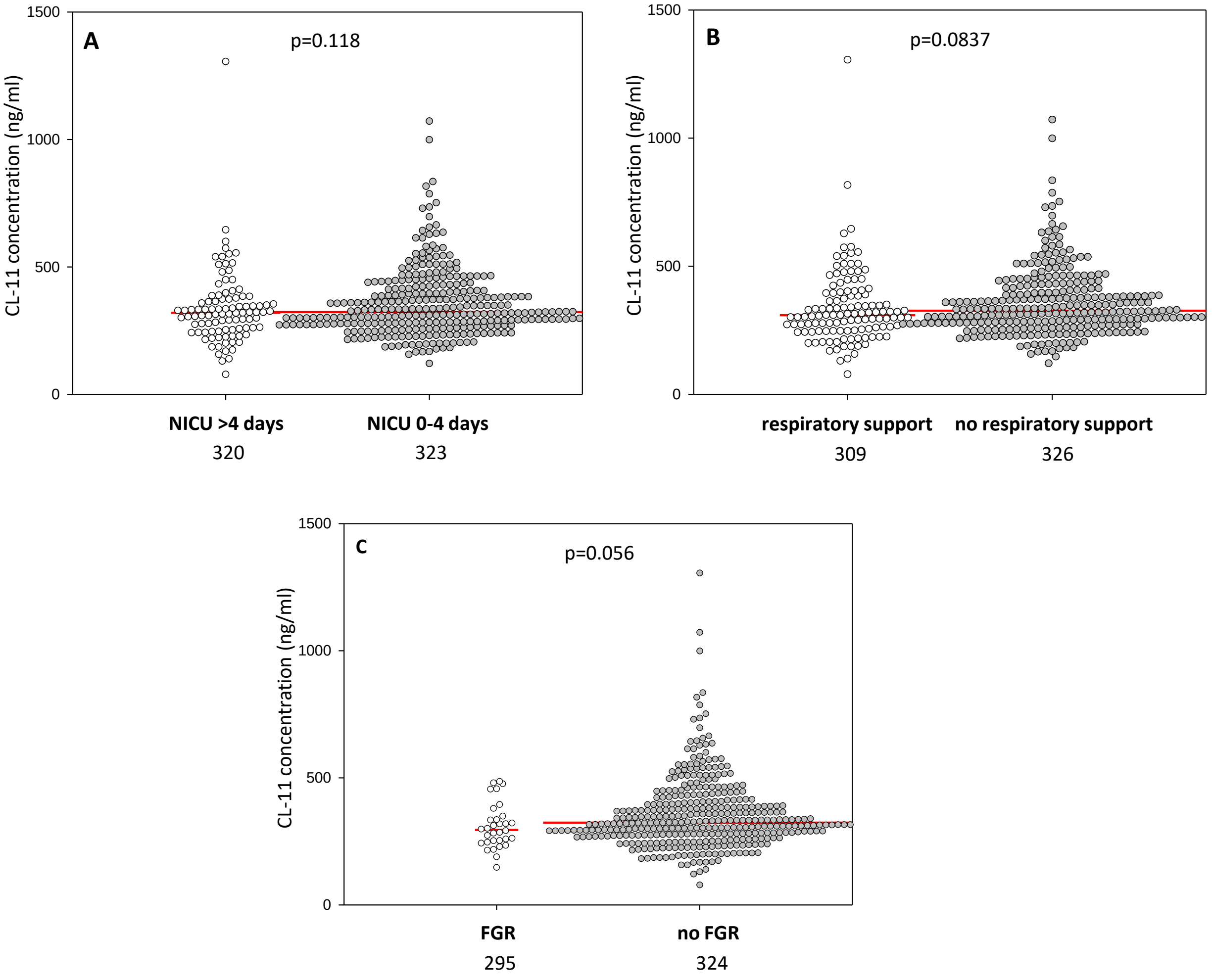

Supplementary Figure 8. Individual concentrations of collectin-11 in preterm neonates, depending on lemght of intensive care (**A**), need for respiratory support (**B**), and incidence of fetal growth restriction (**C**). Red bars represent median values (demonstrated as numbers below the graphs). Statistical significance values show comparisons with the use of Mann-Whitney *U* test.

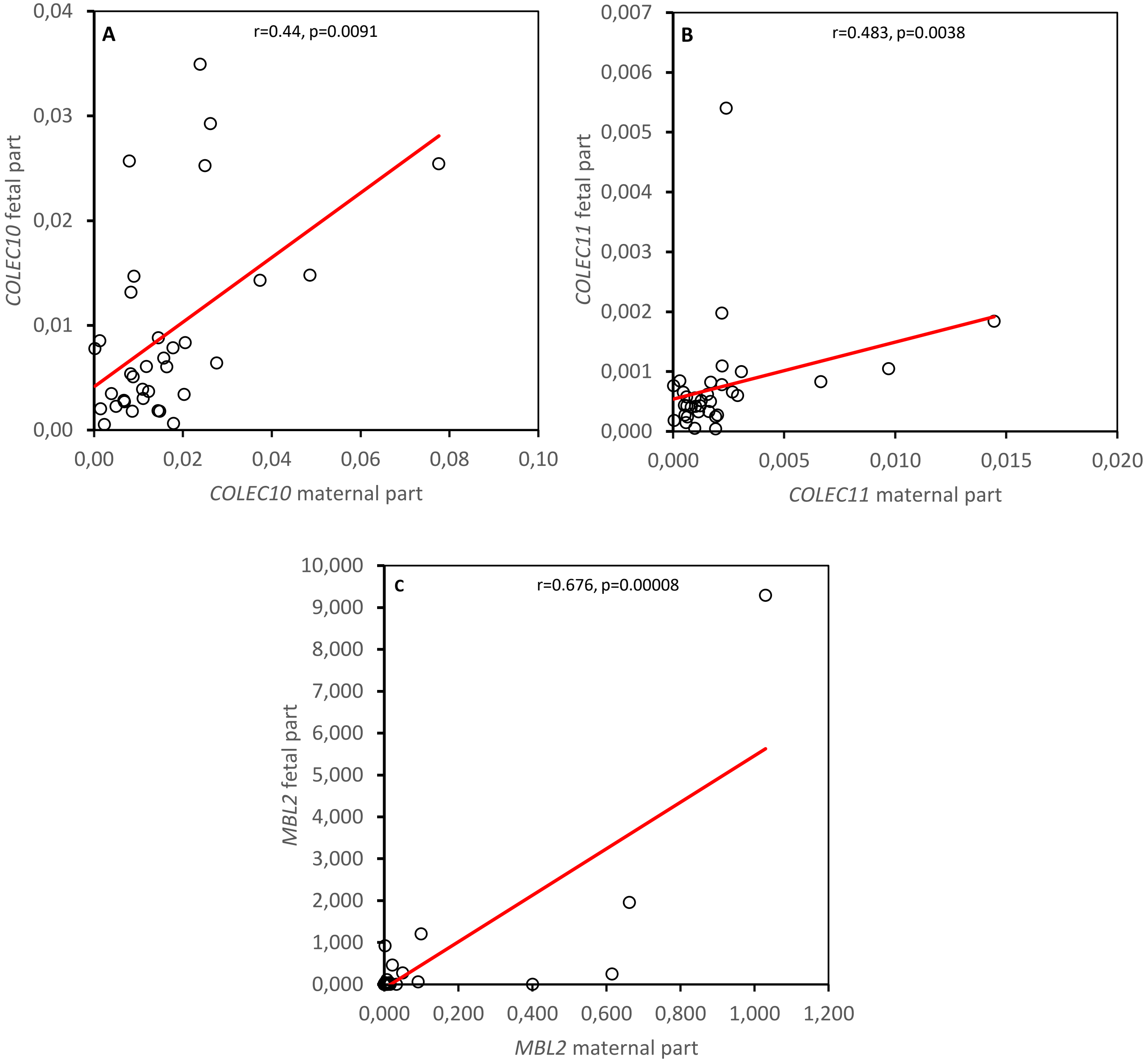

Supplementary Figure 9. Correlations (Spearman) between *COLEC10* (**A**), *COLEC11* (**B**), and *MBL2* (**C**) mRNA expression ($2^{-\Delta Ct}$) in maternal and fetal parts of placentas. Trend lines are shown in red.
